# Structural evolution of microfibers in seawater and freshwater under simulated sunlight: A small- and wide-angle X-ray scattering study

**DOI:** 10.1371/journal.pone.0328502

**Published:** 2025-09-10

**Authors:** Astra Piccinini, Giulia Lucia, Daniele Colarossi, Paolo Principi, Heinz Amenitsch, Lucia Pittura, Francesco Regoli, Stefania Gorbi, Francesco Spinozzi

**Affiliations:** 1 Department of Life and Environmental Sciences, Polytechnic University of Marche, Ancona, Italy; 2 Department of Industrial Engineering and Mathematical Sciences, Polytechnic University of Marche, Ancona, Italy; 3 Institute for Inorganic Chemistry, Graz University of Technology, Graz, Austria; University of Sharjah, UNITED ARAB EMIRATES

## Abstract

Microfibers are pollutants of increasing concern, as they accumulate in aquatic environments and pose risks to living organisms. Once released, they undergo degradation processes that reduce their size and enhance their ability to interact with biological systems. Among these processes, photodegradation is a key driver, leading to fiber fragmentation and structural shrinkage. This study investigates the nanostructural evolution of five common microfibers (cotton, cellulose acetate, polyamide, polyester, and linen) dispersed in seawater and freshwater, following simulated solar ultraviolet exposure equivalent to one year of environmental conditions. Using synchrotron-based Small- and Wide-Angle X-ray Scattering, we characterized changes in the internal organization of the fibers. Small-Angle X-ray Scattering data were analyzed using a multiscale model that describes macrofibrils as bundles of polydisperse core-shell microfibrils arranged in a distorted hexagonal lattice. Results reveal distinct nanostructural modifications. In seawater, cotton and polyamide show increases in microfibril spacing, resulting in macrofibril enlargement, while in freshwater their structure remains more stable or evolves irregularly. Polyester and linen exhibit a progressive reduction in macrofibril diameter in both media, suggesting a greater tendency toward fragmentation. Cellulose acetate remains structurally stable in seawater but undergoes shrinkage and partial reorganization in freshwater. Wide-Angle X-ray Scattering confirms the presence of crystalline phases in cotton, linen, and polyamide, and reveals medium-specific variations in crystallite size, particularly under seawater conditions. These structural differences were quantified using Rietveld refinement of the diffraction data. The structural evolution of microfibers under environmentally relevant conditions has direct implications for their fragmentation potential, persistence, and the release or transport of adsorbed contaminants. The results underscore the importance of considering material-specific behaviors in assessing environmental persistence and potential ecological impact.

## Introduction

Anthropogenic microfibers (MFs), due to their widespread presence in natural environments, have been recognized as emerging pollutants that interact with organisms primarily through ingestion, causing various toxicological effects [1]. Their hazard arises not only from the debris themselves but also from contaminants absorbed from the environment and additives that can leach when MFs enter aqueous systems [2–4]. The bioaccumulation and biomagnification of these compounds may have serious consequences for organisms, particularly at higher food-chain levels [5].

MFs are fibrous materials with diameters below 50 μm and lengths ranging from 1 μm to 5 mm, composed of natural (e.g., cotton, linen, silk, wool), semi-synthetic (e.g., cellulose acetate, rayon), or synthetic polymers (e.g., polyamide, polyester, polypropylene) [6,7]. MFs can be directly used as raw materials in the textile industry or originate from the breakdown of both apparel and non-apparel products, such as cigarette filters, wet wipes, surgical masks, fishing nets and ropes, and tires [8]. MFs pollution can occur throughout the entire life cycle of fibrous materials, from production to disposal, and once released into the environment, both primary and secondary MFs undergo various degradation processes [9–11]. Most MFs found in aquatic ecosystems come from the degradation of textiles and cigarette butts, released either directly into water sources or transported via atmospheric circulation, surface and subsurface runoff, and laundry discharges, as wastewater treatment plants do not fully retain them [12,13].

Degradation of MFs in water involves photodegradation by ultraviolet (UV) solar radiation, thermo-oxidation, hydrolysis, and biodegradation [9]. Although these processes act in synergy to alter the size and structure of MFs, UV-induced photoaging is considered the primary mechanism initiating polymer degradation and the main factor affecting MFs floating on the water surface and in the photic zone. Other abiotic and biological processes instead contribute to MFs weathering in the aphotic zone [14,15]. Photodegradation impacts various materials, including thermoplastic and cellulosic fibers, causing chemical cross-linking or chain scission that leads to morphological changes such as discoloration, surface cracking, yellowing, embrittlement, and fiber weakening [16,17].

The weakening is related to the uniaxial orientation of the polymers in the fibers, which facilitates fragmentation under UV solar radiation [18]. This fragmentation not only generates new MFs but also produces progressively smaller particles, potentially down to the nanometer scale [19]. In aquatic pollution contexts, size reduction increases the hazard of MFs by making them accessible to a wider range of organisms, enhancing their ability to penetrate cells and tissues [20], increasing their sorption capacity for chemical contaminants due to higher surface area [21], and promoting the release of additives, chemical residues, and degradation products [3]. Moreover, changes in size and structural properties induced by photoaging can influence the horizontal and vertical distribution of MFs in aquatic environments, affecting the spatial and temporal patterns of their pollution [22].

Investigating the UV degradation of polymers under natural conditions is impractical and difficult to reproduce. Therefore, accelerated weathering studies simulating environmental conditions are the most commonly used approach to gain a basic understanding of the degradation mechanisms of materials subjected to specific exposure cycles [16]. The chemical-physical techniques most often employed to study the degradation of MFs by UV radiation include rheology, spectroscopy (Fourier transform infrared spectroscopy, FTIR, and X-ray photoelectron spectroscopy, XPS), and microscopy (scanning electron microscopy, SEM, and atomic force microscopy, AFM) [3,15,23–25].

Moezzi *et al*. [24] evaluated the degradation of nylon 66 fabrics under artificial UV exposure using FTIR, XPS, SEM, shear modulus, and thermogravimetric analysis (TGA). They found that the initial decline in mechanical properties, due to polymer chain breakage and the formation of free radicals, was partially recovered after 15 hours of UV exposure. This effect was attributed to the formation of crosslinks between free radicals and a concurrent increase in crystallinity. However, with prolonged UV irradiation, the degrading effects prevail over the bond formation.

Naik *et al*. [26] provided evidence that MFs degeneration from UV exposure depends on the type of plastic: high-density polyethylene and nylon 6 produced microplastic fibers, whereas high-impact polystyrene and polypropylene showed no physical degradation under SEM, appearing more resistant to photo-induced physical changes than the other polymers.

Similarly, Sørensen *et al*. [3] showed that different types of MFs respond differently to UV exposure (300–400 nm, 56 d): polyamide fibers primarily exhibited surface morphology changes, while polyester and wool fibers showed less surface alteration but increased fragmentation into shorter fibers. Additionally, the release of chemicals into the water medium was observed for all aged MFs, regardless of polymer type.

The formation of micro- and nanosized fibers through photo-fragmentation was also reported by Saliu *et al*. [27], following the artificial UV aging (340 nm, 10 hours) of surgical masks. SEM revealed cracks and crazing in the structural fibers of the products, while μFTIR analysis indicated signs of oxidation.

Belzagui *et al*. [13] found that cellulose acetate exhibits very low degradability in aquatic environments. After exposing cigarette butts to UV light (365 nm, 18 months) in both saltwater and distilled water, they highlighted that MFs released from cigarette filters could persist in the environment for extended periods.

More recently, Zambrano *et al*. [25] investigated the effect of UV irradiation (14 d at 254 nm) on polyethylene MFs using FTIR, SEM, and contact angle analysis, showing that UV exposure increased surface roughness and the presence of oxygen-containing functional groups.

While the photochemical degradation and microscopic alterations of MFs have been previously reported, their structural modifications at the nanoscopic scale remain largely unexplored. Moreover, despite growing interest in the environmental implications of MF degradation in aquatic environments, most studies have concentrated on the weathering of plastic polymers in seawater. This focus stems from the longstanding attention to microplastic pollution in marine settings. By contrast, little is known about the effects of UV light on synthetic materials in freshwater, or about the degradation mechanisms affecting natural and semi-synthetic polymers in both freshwater and seawater conditions [7]. These knowledge gaps need to be addressed, especially considering that freshwater environments are polluted by plastic MFs to a degree comparable to marine systems [1], and that different degradation pathways may be expected due to the distinct physicochemical properties of the two environments [15].

Although synthetic polymers currently account for two-thirds of global fibers production, several studies have reported that natural polymers dominate environmental samples. In particular, cellulose-based MFs represent 60–80% of all MFs detected in aquatic environments, and the reasons for their unexpectedly long environmental persistence remain under debate [28].

Building on these premises, this study investigates the structural modifications induced by UV radiation in the most widespread MFs present in aquatic environments. The focus is on textile MFs made of synthetic polymers (polyester and polyamide), natural polymers (cotton and linen), and cellulose acetate derived from cigarette filters. The potential influence of seawater and freshwater conditions on the degradation process was also considered. The primary objective is to characterize changes occurring at the nanometric scale (1–100 nm) by employing two advanced physical techniques: small-angle and wide-angle X-ray scattering (SAXS and WAXS, respectively) [29–31].

SAXS data were analyzed using the GENFIT software [32], by modeling MFs as flexible cylinders (macrofibrils) with a worm-like spatial distribution [33], internally composed of bundles of microfibrils represented as core-shell cylinders with a locally hexagonal arrangement. WAXS data from MFs exhibiting diffraction peaks were analyzed by Rietveld refinement using the GSAS software [34]. The resulting structural insights at the nanometric scale are expected to enhance our understanding of the environmental fate and weathering behavior of some of the most common MFs in both marine and freshwater systems. A table of abbreviations used throughout the manuscript (S1 Table), along with a list of the fiber types investigated in this study (S2 Table), is provided in the Supporting Information.

## Materials and methods

### Sample preparation

Textile MFs made of cotton (length: 400±300 μm; diameter: 16±4 μm), linen (length: 300±200 μm; diameter: 17±5 μm), polyester (length: 600±400 μm; diameter: 13±1 μm) and polyamide (length: 600±500 μm; diameter: 11±1 μm) were supplied by IPCB-CNR (Pozzuoli, Napoli, Italy) which had produced them through micronization of standard fabrics, i.e. materials free of coloring and surface treatments (additional information is available in Pittura *et al*. [35]). Cellulose acetate MFs were instead prepared by using a cryostat to cut non-smoked cigarette butts with 5 μm thickness. For each type of MF, one aliquote was dispersed in 100 mL of artificial seawater (ASW, Instant Ocean Sea Salt at 35 psu) and a second aliquote was dispersed in 100 mL of freshwater (FW, tap water), both filtered at 0.2 μm pore size filters. For all the 10 samples, the w/v MFs concentration was 1 g/L.

### UV irradiation

Water-dispersed MFs were placed in glass bottles with quartz caps (see S1 Fig), a material that does not absorb the UV component of solar radiation (200–400 nm, see S2 Fig). This UV component, primarily responsible for material degradation, is thus allowed to enter the container and reach the fibers. Additionally, all plastic parts of the bottles were carefully covered with aluminum foil to reflect the simulated solar radiation away and to prevent contamination of the samples by plastic degradation residues. To simulate degradation processes occurring in aqueous environments with wave motion, the bottles were placed on a rotating plate. Furthermore, two ventilators were employed to mitigate heat generated by the solar simulator components (S3 Fig). Two additional bottles, filled with 100 mL of ASW and 100 mL of FW respectively, were kept free of fibers and used as controls.

A major challenge in experimentally evaluating the effect of solar radiation on degradation processes is the duration of the tests. Considering the natural day-night cycle and the variable flow conditions throughout the day (e.g., cloud passage) and across seasons, long exposure times are often required to observe significant degradation effects. To overcome this limitation and enable shorter experimental times, solar simulators are commonly used. These devices reproduce natural sunshine approximately with an artificial light source, allowing indoor tests to be programmable, repeatable, and stable under controlled conditions.

For this reason, experiments were conducted under a solar simulator specifically designed with a support structure featuring adjustable positions for a set of lamps that closely reproduce solar radiation (S3A Fig). The lamps used are Osram Ultra-Vitalux 300 W E27, selected for their sun-like radiation spectrum, and arranged in a 4×4 matrix (S3A Fig). The distance between the lamp array and the target area (approximately 30 cm) was set to achieve an irradiance of about 1000 W/m^2^ (S3B Fig), corresponding to the peak solar intensity on a typical sunny summer day in Europe.

To simulate one year of natural exposure under European climate conditions, the total annual UV energy reaching the surface was estimated as 60 kWh/m^2^, approximately 5% of the total solar energy (1200 kWh/m^2^). The UV component of the Osram lamps corresponds well to this value: according to the datasheet, each lamp emits about 16 W of UV radiation out of a total power of 300 W, which is also approximately 5%. With a total flux of 1000 W/m^2^ on the target area, the resulting UV irradiance was 60 W/m^2^. Moreover, considering the height of the glass bottles used in the setup, no correction for depth-dependent attenuation of the incident radiation at the water surface was necessary.

Based on these parameters, the duration of the experimental exposure was set to approximately 42 days. A summary of the relevant physical parameters is provided in S3 Table.

### SAXS and WAXS experiments

SAXS and WAXS experiments were performed at the Austrian SAXS beamline of Elettra synchrotron in Trieste (Italy) [36]. Measurements were carried out at room temperature using the sample holder shown in S4 Fig. The modulus of the scattering vector (q=4πsinθ/λ, 2θ being the scattering angle and λ=1.54 Å the X-ray wavelength) was fixed between 0.01 and 0.4 Å^−1^, for SAXS, and between 1 and 2 Å^−1^ for WAXS. The measured samples were aliquotes of the aqueous dispersions of the ten MFs taken from the glass bottles after being irradiated with the solar simulator for different days (between 0 and 42). For each sample, eighteen SAXS and WAXS frames, each lasting 5 s, were collected, treated with FIT2D [37], and subsequently the data reduction procedure, based on SAXSDOG [38], was applied to subtract either the ASW or FW signal and to obtain the experimental macroscopic differential scattering cross sections $\frac{d\Sigma }{d\Omega }(q)$. No absolute calibration has been performed.

### SAXS and WAXS models

**Fig 1:**
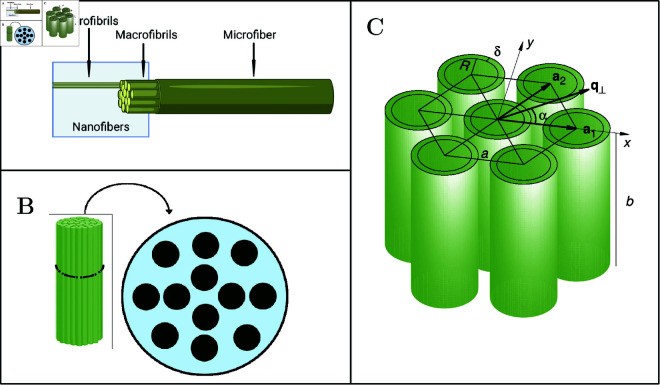
Structural levels of MFs. (A) Structure of one MF; (B) Structure of a macrofibril with several microfibrils enclosed within it; (C) Arrangement of a bundle of parallel core-shell cylinders, with core radius *R*, shell thickness δ, and length *b*, packed in a hexagonal array with lattice parameter *a* and unit vectors $a_{1}$ and $a_{2}$. The two-dimensional vector $q_{⟂}$, in the plane perpendicular to the bundle and forming an angle α with the *x*-axis, is also shown.

MFs exhibit hierarchical structural levels. According to the literature [39], at the first level, they are composed of bundles of *macrofibrils*. At the second level, these macrofibrils are formed by intertwined *microfibrils*, as illustrated in Fig 1A. Both the macrofibrils and the microfibrils, collectively called nanofibers, have nanometric dimensions (1–100 nm), typically analyzed by SAXS/WAXS techniques.

#### SAXS model.

SAXS curves of MFs have been fitted with the worm-like model with excluded volume effects, developed by Pedersen and Schurtenberger [33], combined with the cross section of locally parallel core-shell cylinders [40], representing the microfibrils, organized in a two-dimensional finite and polydisperse hexagonal lattice, with paracrystalline lattice distortion of the second kind [41–43], in order to represent one macrofibril. This model, inspired by the method of Penttilä *et al*. [44], has been implemented in the software GENFIT [32]. In detail, the macroscopic differential scattering cross section is written as

$$\frac{d\Sigma }{d\Omega }(q)=\kappa P_{\text{wl}}(q)P_{\text{cs}}(q)+B$$ (1)

where κ is a scaling factor and *B* is a flat background. They are unimportant but necessary parameters in order to deal with SAXS data in arbitrary unit. $P_{\text{wl}}(q)$ is the form factor of the infinitely thin worm-like chain: it depends on the statistical segment (Kuhn) length *b*, which can be considered the average length of the cylinder bundle (Fig 1C), and by the number of statistical segments *n*_*b*_. Notice that the contour length *L*, which represents the length of the fully extended chain, is simply given by *L* = *n*_*b*_*b*. Since the overall length *L* of the microfibrils is difficult to determine reliably from SAXS data, we have fixed its value at 5000 Å, following the SANS (small-angle neutron scattering) study on cellulose by Martínez-Sanz[45]. As a result, the number of segments is simply obtained as *n*_*b*_ = *L*/*b*, using the statistical segment length *b* as a fitting parameter.

According to the scattering theory of rod-like particles [30], the mean form factor of the macrofibril cross section, $P_{\text{cs}}(q)$, is calculated as the integral, over the angle α around the axis of the cylinder bundle (Fig 1C), of the cross section $P_{\text{cs}}(q_{⟂})$ that depends on the two-dimensional scattering vector in the plane perpendicular to the axis of the macrofibril, $q_{⟂}=(q\cos\alpha ,q\sin\alpha )$, according to

$$P_{\text{cs}}(q)=\frac{1}{2\pi }\int_{0}^{2\pi }P_{\text{cs}}(q_{⟂})d\alpha .$$ (2)

In turn, for an array of parallel core-shell cylinders with polydisperse core radius *R* and shell thickness δ, the function $P_{\text{cs}}(q_{⟂})$, according to the finite paracrystal theory, is

$$P_{\text{cs}}(q_{⟂})=<\;A^{2}(q)\;{>}_{R}+<\;A(q)\;{>}_{R}^{2}[S_{\text{cc}}(q_{⟂})-1]$$ (3)

where *A*(*q*) is the SAXS amplitude corresponding to the cross section of a core-shell cylinder [30,43] and $S_{\text{cc}}(q_{⟂})$ represents the structure factor of a finite and polydisperse paracrystalline hexagonal lattice. Assuming the core consists solely of dry polymeric material and the shell is composed of the same material with a volume fraction ϕ, along with water, the amplitude *A*(*q*) is given by

$$A(q)=2\pi \Delta \rho \left[(1-\phi )R^{2}\frac{J_{1}(qR)}{qR}+\phi (R+\delta {)}^{2}\frac{J_{1}(q(R+\delta ))}{q(R+\delta )}\right]$$ (4)

where *J*_1_(*x*) is the Bessel function of the first kind and Δρ is the difference between the the scattering length density (SLD) of the dry polymer and the one of water. The two averages over the core radius *R* shown in Eq 3 are computed assuming a non-negative Gaussian distribution function defined for $R\geq R_{\text{min}}$, with a maximum at *R*_0_ and with dispersion *g*_*R*_ (standard deviation $\sigma_{R}=g_{R}R_{0}$),

$$<\;A(q)\;{>}_{R}=\int_{R_{\text{min}}}^{\infty }A(q)\;p(R)dR$$ (5)

$$<\;A^{2}(q)\;{>}_{R}=\int_{R_{\text{min}}}^{\infty }A^{2}(q)\;p(R)dR$$ (6)

where the normalized Gaussian distribution is given by

$$p(R)=\frac{e^{-(R-R_{0}{)}^{2}/(2g_{R}^{2}R_{0}^{2})}}{\int_{R_{\text{min}}}^{\infty }e^{-(R-R_{0}{)}^{2}/(2g_{R}^{2}R_{0}^{2})}dR}$$ (7)

In our case we set $R_{\text{min}}=5$ Å. The function *p*(*R*) is then used to compute the mean core radius,

$$<\;R\;>=\int_{R_{\text{min}}}^{\infty }R\;p(R)\;dR$$ (8)

Notice that, since the integral is calculated between $R_{\text{min}}$ and ∞, <R> could be different from *R*_0_. In practice, all the integrals over *R* (Eqs 5–8) are numerically calculated with the Simpson’s rule with 30 points in the interval between $\text{min}\{R_{\text{min}},R_{0}(1-3g_{R})\}$ and $R_{0}(1+3g_{R})$. In Eq 3, the function $S_{\text{cc}}(q_{⟂})$ is computed using the following expression[41–43]:

$$S_{\text{cc}}(q_{⟂})=\sum_{j=-2\sigma_{p}}^{2\sigma_{p}}p_{j}^{2}\prod_{k=1}^{2}S_{\text{PT}}(F_{k},N_{a}+j)$$ (9)

$$S_{\text{PT}}(F_{k},N_{a}+j)=ℜ\left[\frac{1+F_{k}}{1-F_{k}}-\frac{2F_{k}(1-F_{k}^{N_{a}+j})}{(N_{a}+j)(1-F_{k}{)}^{2}}\right]$$ (10)

where $F_{k}=e^{-\frac{1}{2}(ag_{a}q{)}^{2}}e^{iq_{⟂}\cdot a_{k}}$ (*i* is the imaginary unit). The hexagonal unit cell vectors are $a_{1}=a\;\hat{x}$ and $a_{2}=a(\cos\frac{\pi }{3}\;\hat{x}\;+\;\sin\frac{\pi }{3}\;\hat{y})$ (see Fig 1C, where $\hat{x}$ and $\hat{y}$ are unit vectors along *x* and *y* directions).

Relevant parameters of the model, in addition to the lattice length *a*, include the number of lattice units *N*_*a*_ along each of the two directions $a_{1}$ and $a_{2}$, and the so-called distortion parameter *g*_*a*_, defined as $g_{a}=\sigma_{a}/a$, where $\sigma_{a}$ is the Gaussian standard deviation associated with the isotropic distortion of the paracrystal. Note that, within this framework, the macrofibril diameter is estimated as $D=a(N_{a}\;-\;1)$. To suppress the intrinsic oscillations observed in the monodisperse paracrystalline structure factor at low *q*, which are never seen in experimental data [42], polydispersity over *N*_*a*_ must be introduced. Sampling points are weighted according to a discrete Gaussian distribution,

$$p_{j}=\frac{e^{-\frac{1}{2}j^{2}/\sigma_{p}}}{\sum_{j^{\prime }=-2\sigma_{p}}^{2\sigma_{p}}e^{-\frac{1}{2}j^{\prime 2}/\sigma_{p}^{2}}}$$ (11)

with standard deviation

$$\sigma_{p}=\left\{ \begin{array}{cc} \frac{1}{2}(N_{a}-1) & N_{a}<5\\ N_{a}^{1/2} & N_{a}\geq 5 \end{array}\right.$$ (12)

#### WAXS model.

The isotropic WAXS data showing diffraction peaks were analyzed using GSAS (General Structure Analysis System), a well-established software package developed by Larson and Von Dreele for the analysis of X-ray and neutron diffraction data [46]. It enables crystallographic refinement based on Rietveld analysis of powder diffraction patterns. GSAS is commonly used in combination with EXPGUI, a graphical user interface that simplifies model setup, parameter control, and visualization of results [47]. A notable feature of GSAS is its ability to import structural models from Crystallographic Information Files (CIFs), which contain atomic coordinates, unit cell parameters, and symmetry information. Background modeling in GSAS can be performed using shifted Chebyshev polynomial functions. This approach allows for flexible modeling of diffuse scattering or instrumental background across the diffraction pattern, improving the accuracy of peak intensity determination. For peak shapes and widths, GSAS provides several profile functions based on pseudo-Voigt formulations, allowing for the modeling of both Gaussian and Lorentzian components of the diffraction peaks. The peak width is modeled as a function of the scattering angle (2θ), incorporating contributions from both instrumental resolution and crystallite size. In this work, instrumental broadening was characterized by measuring a *p*-bromo benzoic acid standard and deriving the instrument parameter file from its known diffractogram. This file was then imported into GSAS. The refinement procedure included the unit cell parameters ($a_{\text{cel}}$, $b_{\text{cel}}$, $c_{\text{cel}}$, and the angles $\alpha_{\text{cel}}$, $\beta_{\text{cel}}$, and $\gamma_{\text{cel}}$), as well as the Lorentzian broadening $\sigma_{\text{L}}$ (in degrees), which was used to calculate the crystallite size, according to

$$D_{c}=\frac{180\;k\lambda }{\pi \sigma_{\text{L}}}$$ (13)

where k≈0.9 is the Scherrer constant. The diffuse background was fitted using five shifted Chebyshev functions of the first kind.

## Results and Discussions

SAXS and WAXS experiments were carried out at the Elettra synchrotron on five types of MFs, each dispersed in either ASW or FW. To assess the effect of UV radiation, each of the ten samples was exposed to a solar simulator for 42 d, corresponding to the UV dose expected from one year of natural sunlight exposure.

The SAXS data were analyzed using the GENFIT software [32]. This model determines the core radius of the internal microfibrils, treated as a polydisperse parameter following a non-negative Gaussian distribution characterized by a maximum (*R*_0_), a dispersion (*g*_*R*_), and a mean value (<R>). The thickness of the cylindrical shell surrounding each microfibril (δ) and the polymer volume fraction within the shell (ϕ) are also estimated. Furthermore, the model provides the average number of microfibrils across the macrofibril diameter (*N*_*a*_), their average spacing (*a*), and the degree of disorder (*g*_*a*_) in the local hexagonal arrangement formed by the projection of microfibril axes on the macrofibril cross-section (Fig 1C). From these parameters, the macrofibril diameter (*D*) can be calculated. The average length of the rigid segments (*b*) is also estimated, from which the total number of segments (*n*_*b*_) is obtained, given the fixed length of the extended macrofibril (*L*). To quantify the uncertainty of all model fitting parameters, the fit was repeated 20 times using data resampled from normal distributions defined by the experimental uncertainties. For the sake of completeness, correlation matrices among the fitted parameters for the SAXS data at *t* = 0 d are reported in S4–S13 Tables, for each fiber type and water medium. These matrices indicate generally low correlations, not exceeding an absolute value of ≈0.3.

WAXS data displaying distinct diffraction peaks were analyzed with the GSAS software [46], using the available CIF file of the polymer crystals. Fitting parameters include the unit cell dimensions ($a_{\text{cel}}$, $b_{\text{cel}}$, $c_{\text{cel}}$) and angles ($\alpha_{\text{cel}}$, $\beta_{\text{cel}}$, $\gamma_{\text{cel}}$), from which the unit cell volume ($V_{\text{cel}}$) is derived. The Lorentzian width ($\sigma_{\text{L}}$) is employed to estimate the crystallite size (*D*_*c*_). Uncertainties on the refined parameters were obtained from the inverted least-squares matrix provided by GSAS, allowing a quantitative evaluation of their accuracy.

**Fig 2:**
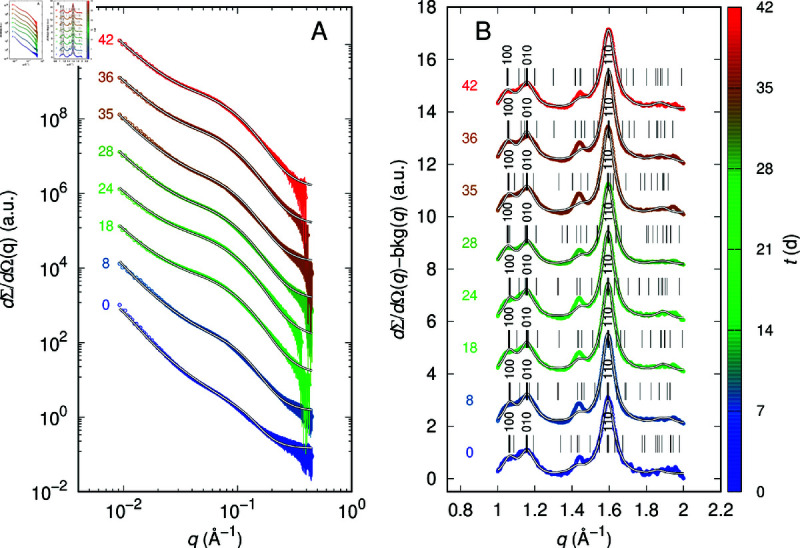
SAXS and WAXS curves of cotton MFs in ASW. The color of the data points corresponds to the vertical color scale, which indicates the UV irradiation time. A) The solid black and white lines represent the best fits obtained using GENFIT. Starting from the bottom, each curve is multiplied by a factor of 10 relative to the one below it, for clarity. B) The black and white lines correspond to the best fits obtained using GSAS. The experimental curves (shown without error bars for clarity) and their respective fits are presented, with the diffuse background, determined from the fit, subtracted from each. Thin vertical black lines indicate the Bragg reflections of the space group. Thick vertical black lines, with the corresponding Miller indices indicated, mark the positions of the main peaks. The curves are vertically offset by a factor of 2 for clarity.

**Fig 3:**
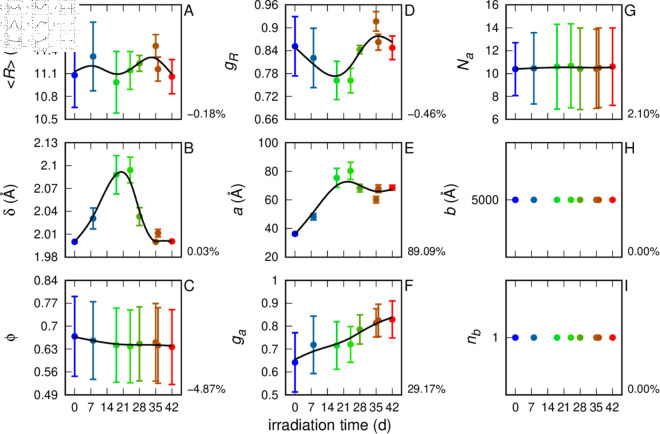
Fitting parameters from SAXS analysis of MFs of cotton in ASW. Smooth black curves among the points have been obtained with cubic splines weighted with uncertainties of the parameters. In the lower right corner of each panel, the percentage change between *t* = 0 d and *t* = 42 d, relative to the initial value, is reported.

**Fig 4:**
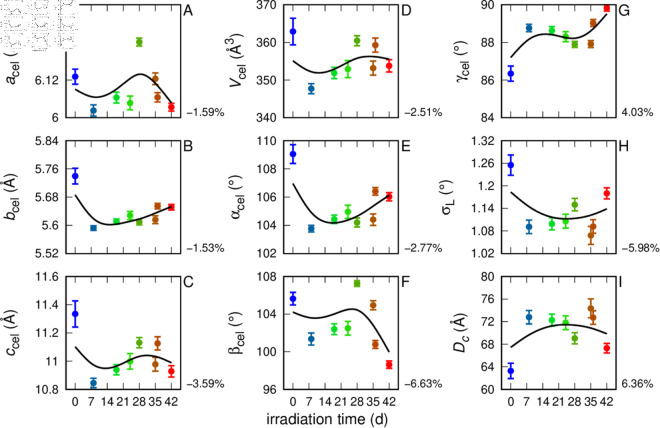
Fitting parameters from WAXS analysis of MFs of cotton in ASW. Smooth black curves among the points have been obtained with cubic splines weighted with uncertainties of the parameters. In the lower right corner of each panel, the percentage change between *t* = 0 d and *t* = 42 d, relative to the initial value, is reported.

The following sections present and discuss the results for each of the five MFs, comparing their behavior in ASW and FW. For cotton MFs in ASW, the experimental and fitted SAXS and WAXS curves are shown in Fig 2 (panels A and B, respectively). Corresponding fitting parameters plotted versus UV irradiation time are detailed in Figs 3 and 4. Data for the other samples, including experimental and fitted curves and parameters, are available in S5–S27 Figs.

**Fig 5:**
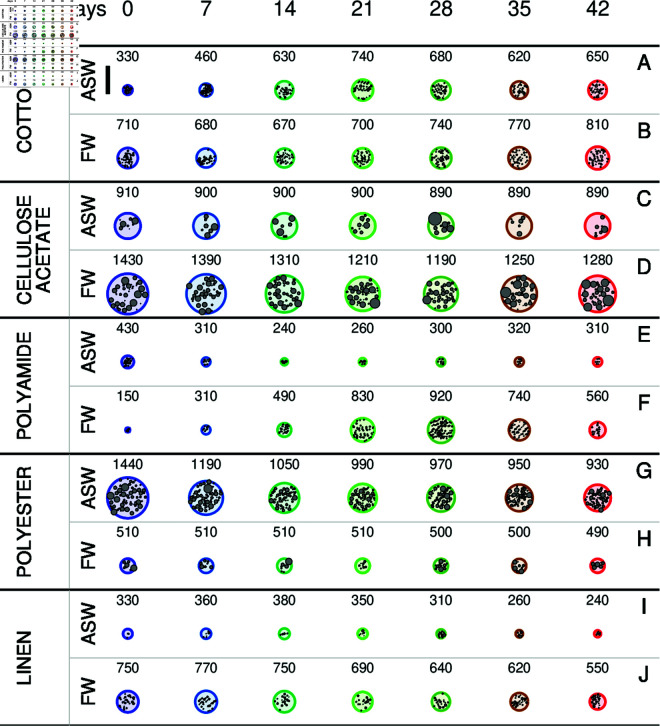
Cross-sections of macrofibrils during UV irradiation. Representation of the structure of the macrofibril’s circular cross section (large colored circles) as a function of the UV irradiation time according to the SAXS fitting parameters for the five investigated MFs in ASW (panels A, C, E, G and I) and in FW (panels B, D, F, H, J). The diameter *D* of the macrofibril’s circular cross section has been selected on the basis of the fitting parameters *a* and *N*_*a*_, according to $D=a(N_{a}\;-\;1)$. Its value is indicated in Å at the top of each image. The small gray circles inside the large circles, representing the microfibrils, have been traced with a core radius *R* sampled by the Gaussian distribution *p*(*R*) (Eq 7) using the fitting parameters *R*_0_ and *g*_*R*_. The positions of their centres have been sampled from a distorted hexagonal lattice, following the paracrystal theory, using the fitting parameters *a*, *g*_*a*_, and *N*_*a*_. The thick vertical black line on the left of panel A represents the length of 1000 Å.

A visualization of the macrofibrils’ circular cross-sections, including their internal microfibrils, was constructed based on SAXS fitting parameters across the ten MFs and irradiation times (Fig 5). The microfibrils (small gray circles) within the larger macrofibrils (colored circles) are shown with radii sampled from the fitted Gaussian polydisperse distribution. Their positions are sampled according to the bidimensional paracrystal parameters *a*, *g*_*a*_, and *N*_*a*_. The vertical black line to the left of panel A represents 1000 Å. A detailed view of the microfibril core-shell structure is provided in S29 Fig. Finally, the non-negative Gaussian distributions of the core radius are shown in S30 Fig.

### Cotton

SAXS curves for cotton MFs in ASW are presented in Fig 2A. The observed patterns closely resemble those previously reported in several papers by Martínez-Sanz *et al*. [40,45,48,49]. The GENFIT fitting results (Fig 3) reveal a significant increase of approximately 90% in the parameter *a*, which corresponds to the average spacing between microfibrils. Indeed, *a* increases from ≈30 Å at the beginning of the irradiation to a maximum of ≈80 Å around the 21^st^ day (Fig 3E), whereas *N*_*a*_ remains mostly constant at ≈10 (Fig 3G). In contrast, both the microfibrils’ mean core radius <R> (Fig 3A) and the shell thickness δ (Fig 3B) are almost constant and the length of the statistical segment *b* (Fig 3H) reaches the maximum allowed value of 5000 Å, suggesting that the microfibrils behave as rigid rods. To note, the mean core radius is ≈11 Å, in very good agreement with results obtained by Martínez-Sanz *et al*. [40] from SAXS and SANS data of cellulose microfibrils in mature cotton fibres. Basically, the irradiation of the solar simulator in ASW brings the cotton microfibrils to get away from each other and consequently the macrofibril diameter *D* increases from ≈300 Å from ≈700 Å, as it can be easily observed in Fig 5A. The increase of the permeation of water inside the macrofibril is also reflected in the small decrease (in the order of 5%) of the volume fraction ϕ of the polymer in the shell (Fig 3C). In FW conditions (S6A and S7 Figs) the results are quite different: the distance between microfibrils fluctuates between 60 Å to 80 Å throughout the irradiation period, with no significant changes in other parameters. This indicates that UV irradiation does not have a notable effect on the degradation process of cotton MFs in FW (see also Fig 5B).

WAXS results in ASW and FW are shown in Fig 2B and S6B Fig, respectively. According to the literature [50–53], cellulose consists of two distinct crystalline phases: one triclinic (*P*1, I_*α*_) and the other monoclinic (*P*2_1_, I_*β*_). CIF files for both phases were downloaded from the COD database [54] and imported into the GSAS software. Following French [55], the unit cell parameters reported by Nishiyama *et al*. [51] were modified by adopting a convention in which the $c_{\text{cel}}$-axis is aligned with the molecular axis. All fits obtained with GSAS, even when assuming the presence of both phases, assigned a weight to the I_*β*_ phase below the detection limit. Consequently, the fits led to the refinement of the unit cell parameters for the triclinic I_*α*_ phase reported by Nishiyama *et al*. [51] ($a_{\text{cel}}=6.72$ Å, $b_{\text{cel}}=5.96$ Å, $c_{\text{cel}}=10.40$ Å, $\alpha_{\text{cel}}=118.08^{\circ }$, $\beta_{\text{cel}}=114.80^{\circ }$, $\gamma_{\text{ cel}}=80.37^{\circ }$), as well as the determination of the Lorentzian broadening parameter $\sigma_{\text{L}}$. The fitted WAXS curves (Fig 2B and S6 FigB, black/white solid lines) show good agreement with the experimental data. In particular, the main peak (with Miller indices 110) as well as the other two peaks (with indices 100 and 010) are well reproduced. Fitting parameters are plotted as a function of irradiation time in Fig 4 for cotton in ASW and in S8 Fig for the FW case. The average unit cell parameters obtained from the fits of all UV-irradiated samples in the ASW case are: $a_{\text{cel}}=6.09\;\pm \;0.02$ Å, $b_{\text{cel}}=5.64\;\pm \;0.02$ Å, $c_{\text{cel}}=11.03\;\pm \;0.05$ Å, $\alpha_{\text{cel}}=105.4\;\pm \;0.6^{\circ }$, $\beta_{\text{cel}}=102.9\;\pm \;0.9^{\circ }$, $\gamma_{\text{cel}}=88.3\;\pm \;0.3^{\circ }$. These values are very similar to those obtained in the FW case: $a_{\text{cel}}=6.11\;\pm \;0.03$ Å, $b_{\text{cel}}=5.61\;\pm \;0.02$ Å, $c_{\text{cel}}=11.05\;\pm \;0.09$ Å, $\alpha_{\text{cel}}=104.6\;\pm \;0.5^{\circ }$, $\beta_{\text{cel}}=102\;\pm \;1^{\circ }$, $\gamma_{\text{cel}}=89.0\;\pm \;0.4^{\circ }$. However, by examining the trends shown in Fig 4 (case ASW), it can be observed that for almost all parameters there is a marked variation between their values at time *t* = 0 d and those at later times, which appear largely consistent with each other starting from *t* = 7 d. In particular, as shown in the bottom-right panels of Fig 4, the percent differences between the initial and final values of the parameters $\beta_{\text{cel}}$, $\gamma_{\text{cel}}$, $\sigma_{\text{L}}$ and *D*_*c*_ are −6.63%, 4.03%, −5.98% and 6.36%, respectively. By contrast, in the case of FW (S8 Fig), the corresponding percent variations are almost negligible (0.26%, −0.04%, 1.52% and −1.50%, respectively). It is interesting to note that in the presence of ASW, after 7 d of irradiation, the average crystallite size increases from ≈60 Å to ≈70 Å (Fig 4I), and over approximately the same time interval, a significant change is observed in the average distance *a* between microfibrils (Fig 3E), leading to an increase in the average diameter of the macrofibrils (Fig 5A). This phenomenon does not occur in the case of cotton MFs in FW (Fig 5B). Finally, it is worth noting that, in both ASW and FW, for all irradiation times, the average crystallite size is greater than the average diameter of the microfibrils (2<R>), a result that suggests an anisometric shape of the crystallites, with a larger dimension along the microfibril axis than in the perpendicular direction.

### Cellulose Acetate

SAXS curves of cellulose acetate MFs in ASW (S9A Fig) were successfully fitted across the entire *q*-range using GENFIT. The fitting results, shown in S10 Fig, reveal a relatively large initial mean core radius of the microfibrils, <R>=53.6±0.7 Å, which undergoes a slight decrease with increasing irradiation time, reaching 52.3±0.7 Å at *t* = 42 d. The total variation remains limited to −2.51%, indicating a modest structural evolution under UV exposure. The core radius dispersion (*g*_*R*_, S10D Fig) is very high, with an average value of ≈1, resulting in a broad probability density function *p*(*R*) (S30C Fig) that extends up to R≈120 Å. The microfibril spacing (*a*, S10E Fig) fluctuates around 156 Å, the number of lattice units (*N*_*a*_, S10G Fig) shows a slight decrease from the initial value of 6.8, while the distortion parameter (*g*_*a*_, S10F Fig) exhibits a mild increase, with values around 0.7. As a result, the macrofibril diameter (Fig 5C) under UV irradiation changes only slightly, from 910 Å to 890 Å, suggesting that cellulose acetate in ASW maintains the stability of the MF structure, with no significant UV-induced modification observed. In contrast, when cellulose acetate is dissolved in FW (SAXS curves in S11A Fig, and fitting parameters plotted in S12 Fig), a different UV effect is observed. The mean core radius of the microfibrils, <R>, undergoes a significant decrease from ≈60 Å at *t* = 0 d to ≈50 Å at *t* = 21 d, and then increases again to ≈55 Å (S12A and S29D Figs). A similar trend is followed by the core radius dispersion parameter *g*_*R*_ (S12D Fig), resulting in systematic variations in the core radius probability density function *p*(*R*) (S30D Fig). The lattice parameter *a* (S12E Fig) displays a comparable behavior, starting from ≈115 Å, decreasing to ≈104 Å on the 21^st^ day, and increasing back to ≈110 Å at the end of the UV irradiation period. The distortion parameter *g*_*a*_ (S12F Fig) follows a similar evolution, with values of ≈0.34, ≈0.44, and ≈0.38 at *t* = 0 d, 21 d, and 42 d, respectively. The combined effect of these parameters is reflected in the time evolution of the macrofibril diameter *D* (Fig 5D), which is 1430 Å at the beginning of the UV irradiation process, reaches a minimum of 1210 Å at *t* = 21 d, and increases to 1280 Å by the end of the UV irradiation time.

The WAXS curves, shown in S9B and S11B Figs, do not exhibit any diffraction peaks, indicating the absence of crystalline domains in the cellulose acetate.

In summary, cellulose acetate in ASW shows stable structural parameters under UV irradiation, whereas in FW the same treatment leads to pronounced changes in micro- and macrostructural parameters, including a significant reduction and partial recovery of the macrofibril diameter.

### Polyamide

SAXS curves of polyamide (nylon 6) MFs in ASW are shown in S13A Fig. The trends of these curves are consistent with SAXS experiments conducted by Pleštil *et al*. [56], as well as with the SANS and SAXS data reviewed by King *et al*. [57], all of which display a broad scattering bump around q≈0.06 Å^−1^. Fitting parameters obtained with GENFIT are reported in S14 Fig. All parameters clearly undergo a continuous variation as a function of UV irradiation time. The mean core radius of the microfibrils (<R>, S14A Fig) varies by −6.34%, decreasing from 13.8±0.2 Å to 13.0±0.2 Å over a period of 21 d. The core radius dispersion parameter *g*_*R*_ remains quite low, staying approximately constant at 0.26 (S14D Fig). As a result, the probability density functions *p*(*R*) describing the distribution of microfibril radii (S30E Fig) are very similar and concentrated below 24 Å. The thickness of the cylindrical shell changes by −4.61%, starting from an initial value of 6.4 Å (S14B Fig). It appears to be water-rich, with a polymer volume fraction (ϕ) of about 0.2, which is only slightly affected by the irradiation dose (S14C Fig). The lattice parameter *a*, although subject to fluctuations, shows an overall increasing trend, rising from approximately 64 Å to 80 Å, corresponding to an increase of 9.00% (S14E Fig). No significant changes are observed in the distortion parameter *g*_*a*_ (S14F Fig). The number of lattice units *N*_*a*_ decreases from about 8 at *t* = 0 d to around 4 at *t* = 7 d, remaining constant thereafter (S14G Fig). This results in a significant change in the mean macrofibril diameter *D* (Fig 5E), which decreases from 430 Å to approximately 300 Å within 7 d. It is also observed that the length of the rigid segment (*b*, S14H Fig) is relatively low, on the order of 50 Å, with a slight increase over the irradiation time. This parameter is reflected in the number of rigid segments *n*_*b*_ (S14I Fig), which is approximately 95 and does not show significant variation with time.

The WAXS profiles for polyamide (nylon 6) in ASW are displayed in S13B Fig. As widely documented in the literature [58–64], nylon 6 crystallizes primarily into a monoclinic phase (*P*2_1_), referred to as the α phase, which is predominant under standard conditions. A second monoclinic form, the γ phase, is also known to develop, though it is typically associated with high-temperature environments. CIF files for both polymorphs were built using the atomic coordinates reported by Holmes *et al*. [58] and Arimoto *et al*. [59], respectively, and subsequently analyzed using GSAS. All GSAS-based refinements attributed to the γ phase an intensity contribution below the detection threshold. As a result, the refinement procedure yielded unit cell parameters for the α phase consistent with the values reported by Holmes *et al*. [58] ($a_{\text{cel}}=9.56$ Å, $b_{\text{cel}}=17.24$ Å, $c_{\text{cel}}=8.01$ Å, $\alpha_{\text{cel}}=90.0^{\circ }$, $\beta_{\text{cel}}=67.5^{\circ }$, $\gamma_{\text{cel}}=90.0^{\circ }$), and also allowed for the estimation of the Lorentzian broadening parameter $\sigma_{\text{L}}$. The calculated WAXS curves (black/white solid lines in Fig 2B) are in good agreement with the experimental data. Notably, the most intense reflections (indexed as 200 and 002) are well reproduced. The evolution of fitting parameters of the WAXS curves with the UV irradiation time for polyamide in ASW is summarized in the nine panels of S15 Fig. The unit cell dimensions, averaged across all UV-irradiated specimens, are: $a_{\text{cel}}=9.598\;\pm \;0.006$ Å, $b_{\text{cel}}=17.94\;\pm \;0.05$ Å, $c_{\text{cel}}=8.268\;\pm \;0.006$ Å, and $\beta_{\text{ cel}}=66.78\;\pm \;0.09^{\circ }$, with values closely matching those of the CIF model derived from the data by Holmes *et al*. [58]. No appreciable trends are detected in these parameters over the course of irradiation: the largest deviation, observed for $\beta_{\text{cel}}$, amounts to only −1.33% (S15F Fig). Among the fitting parameters, only the Lorentzian broadening $\sigma_{\text{L}}$ exhibits a more marked variation, reaching up to −5.98% (S15H Fig), although the corresponding data points display some dispersion. This decrease is reflected in the evolution of the average crystallite size, which increases from 51.1±0.3 Å at *t* = 0 d to 87.0±0.9 Å at *t* = 42 d, corresponding to a substantial change of 70.10% (S15I Fig), mainly occurring after day 21. As in the case of cotton MFs, a comparison between the crystallite sizes and the average microfibril core radius suggests that the crystallites exhibit an anisometric shape, being more extended along the microfibril axis.

SAXS curves of polyamide in FW under different UV irradiation doses are shown in S16A Fig. Qualitatively, the SAXS curves resemble those obtained in ASW, typically displaying the characteristic bump at q≈0.06 Å^−1^ in most cases. Examining the fit parameters (S14 Fig), we first observe that the average core radius of the microfibrils remains fairly constant, around ≈10 Å (S14A Fig). The distortion parameter of the core radius (*g*_*R*_, S17D Fig) increases by 14.32%, from 0.38±0.03 at *t* = 0 d to 0.43±0.04 at *t* = 42 d. As a result, the probability density functions (*p*(*R*), S30F Fig), which are all confined to R≈24Å, vary with UV irradiation time. The shell thickness δ (S17B Fig) changes with increasing UV dose from 6.7±0.6 Å to 5.1±0.3 Å, corresponding to a percentage variation of −23.34%. Likewise, the polymer fraction in the shell domain (ϕ, S17C Fig) changes from 0.7±0.1 to 0.6±0.2, suggesting a higher level of hydration. The cell parameter *a* (S17E Fig) exhibits a fluctuating trend, reflecting a high degree of sample heterogeneity. As for the distortion parameter of the two-dimensional paracrystal (*g*_*a*_, S17F Fig) and the number of unit cells (*N*_*a*_, S17G Fig), both show a maximum at *t* = 28 d, followed by a return to their initial values, a pattern that may indicate a critical response to the UV dose. As a result, the time dependence of the macrofibril diameter *D*, shown in Fig 5F, is rather peculiar: it starts at 150 Å at *t* = 0 d, reaches a peak of 920 Å at *t* = 28 d, and then decreases to 560 Å at *t* = 42 d. In contrast, the trend for the rigid segment length (*b*, S17H Fig) is more clearly defined: it increases gradually from 43±3 Å to 59±3 Å, with an overall percentage change of 37.97%.

WAXS results of polyamide in FW (S16B and S18 Fig) partially agree with those obtained from SAXS. On one hand, the unit cell parameters (S18A–S18F Fig), averaged over the UV irradiation time, are $a_{\text{cel}}=9.62\;\pm \;0.06$ Å, $b_{\text{cel}}=17.5\;\pm \;0.1$ Å, $c_{\text{cel}}=8.20\;\pm \;0.05$ Å, and $\beta_{\text{cel}}=66.8\;\pm \;0.8^{\circ }$, in agreement with the values found in ASW (S15A–S15F Fig). On the other hand, as highlighted by the smooth solid black lines in the nine panels of S18 Fig, all parameters exhibit a critical trend (either a maximum or a minimum) occurring around *t* = 21−28 d, similarly to what is observed in the SAXS results. In particular, the crystallite size (*D*_*c*_, S18I Fig) starts at ≈60 Å, reaches a minimum of ≈60 Å at t≈28 d, and then increases to ≈80 Å at *t* = 42 d.

### Polyester

SAXS curves of polyester in ASW (S19A Fig) were successfully fitted using GENFIT. Examining the trends of the fitting parameters (S20 Fig), we observe a gradual decrease in the mean microfibril core radius (S20A Fig), from 50±10 Å to 38±6 Å over a period of 42 d (percentage variation −17.43%). The dispersion parameter of the core radius (*g*_*R*_, S20D Fig) is high and progressively increases from 0.93±0.09 to 1.13±0.09, corresponding to a variation of 20.55%. As a result, a broad probability density function *p*(*R*) is obtained (S30G Fig), extending up to R≈120 Å. Smooth trends are also observed for the lattice parameter *a* (S20E Fig), decreasing from 90±20 Å to 60±10 Å, for the lattice distortion parameter *g*_*a*_ (S20F Fig), decreasing from 0.39±0.02 to 0.33±0.01, and for the number of unit cells *N*_*a*_ (S20G Fig), decreasing from 18±1 to 16±1. These variations result in a gradual decrease of the macrofibril diameter *D* (Fig 5G), from 1440 Å at *t* = 0 d to 930 Å at *t* = 42 d. Notably, the length of the rigid statistical segment *b* reaches the upper allowed bound of 5000 Å (S20H Fig), indicating the stiffness of the microfibrils.

SAXS results in FW (curves in S21A Fig and GENFIT fitting parameters in S22 Fig) show similarly gradual parameter variations as a function of UV irradiation time. Regarding the microfibril structure, the mean core radius (<R>, S22A Fig) changes slightly by −4.87%, from 43.5±0.9 Å at *t* = 0 d. The dispersion parameter *g*_*R*_ remains high and nearly constant (S22D Fig), resulting in a broad and UV-dose-independent probability density function *p*(*R*) (S30H Fig). The statistical segment length (5000 Å, S22H Fig) confirms, as in the ASW case, the stiffness of the microfibrils. The lattice parameter *a* and the distortion parameter *g*_*a*_ show slight decreases from their initial values (95±1 Å and 0.59±0.03, respectively, S22E Fig–S22F Fig). Conversely, the number of unit cells remains quite low and nearly constant at $N_{a}\approx 6$ (S22G Fig). Altogether, these results correspond to small macrofibril diameters (*D*, Fig 5 H), varying only slightly from 510 Å to 490 Å between *t* = 0 d and *t* = 42 d.

As in the case of cellulose acetate, WAXS curves for both ASW and FW do not exhibit diffraction peaks (S19B and S21B Fig).

### Linen

SAXS curves of linen MFs in ASW (S23A Fig) and in FW (S26A Fig) resemble those of cotton (Fig 2A and S6A Fig), as expected given that linen fibres are also composed of cellulose. Fitting parameters obtained in ASW with GENFIT (S24 Fig) indicate that the mean core radius of the microfibrils (<R>, S24A Fig) is similar to that of cotton MFs, around ≈11 Å, and slightly increases with UV dose (percentage variation 2.00%). The probability density function *p*(*R*) (S30I Fig) extends up to ≈30 Å, reflecting the relatively high dispersion of the core radius (*g*_*R*_, S24D Fig), approximately 1 and increasing slightly under UV exposure (percent increase of 13.59%). A thin shell thickness is found (δ≈2 Å, S24B Fig), with a high water content (ϕ≈0.1, S24C Fig). The lattice parameter *a* (S24E Fig) undergoes a notable decrease from 76±3 Å at *t* = 0 d to 37±1 Å at *t* = 42 d (percentage change −51.36%), a behavior opposite to that observed in cotton MFs in ASW (Fig 3E). The number of unit cells (*N*_*a*_, S24F Fig) is low but increases with UV dose, from 5±2 to 8±3. As with cotton, the statistical segment length *b* reaches the maximum allowed value of 5000 Å, indicating stiff microfibrils. The combination of these parameters results in a progressive decrease in macrofibril diameter over time (*D*, Fig 5I), from 330 Å to 240 Å.

GENFIT analysis of the SAXS curves of linen MFs in FW (curves in S26A Fig, fitting parameters in S27 Fig) reveals structural similarities as well as differences. The mean core radius remains small, around ≈11 Å, and slightly increases with UV exposure (<R>, S27A Fig). The corresponding probability density function *p*(*R*) (S30J Fig) again extends up to ≈30 Å, due to a high and increasing core radius dispersion (*g*_*R*_, S10D Fig) over the irradiation time. The initial value of the lattice parameter *a* (S27E Fig) is higher than that of linen MFs in ASW (95±2 Å), decreasing to 74±2 Å at *t* = 42 d. This difference, combined with a slightly higher and nearly constant number of unit cells ($N_{a}\approx 8$, S27F Fig), results in larger macrofibril diameters (*D*, Fig 5J), which decrease from 750 Å to 550 Å over time.

WAXS curves of linen MFs in both ASW (S23B Fig) and FW (S26B Fig) closely resemble those of cotton (Fig 2B and S6B Fig, respectively), confirming the presence of cellulose crystals. The analysis of WAXS curves with GSAS, performed following the same procedure used for cotton MFs, confirmed the presence of the triclinic phase (*P*1, I_*α*_) reported by Nishiyama *et al*. [51]. The average refined unit cell parameters (S25A–S25G Fig), obtained from the fits of all UV-irradiated samples in ASW, are: $a_{\text{cel}}=6.06\;\pm \;0.02$Å, $b_{\text{cel}}=5.586\;\pm \;0.009$Å, $c_{\text{cel}}=10.94\;\pm \;0.04$Å, $\alpha_{\text{cel}}=104.1\;\pm \;0.3^{\circ }$, $\beta_{\text{cel}}=101.9\;\pm \;0.7^{\circ }$, $\gamma_{\text{cel}}=88.6\;\pm \;0.2^{\circ }$. Comparable mean values were obtained in the FW case (S28A–S28G Fig): $a_{\text{cel}}=6.20\;\pm \;0.03$Å, $b_{\text{cel}}=5.62\;\pm \;0.03$Å, $c_{\text{cel}}=11.3\;\pm \;0.2$Å, $\alpha_{\text{cel}}=105.4\;\pm \;0.9^{\circ }$, $\beta_{\text{cel}}=106\;\pm \;1^{\circ }$, $\gamma_{\text{cel}}=87.2\;\pm \;0.5^{\circ }$. The only relevant difference between the ASW and FW cases concerns the crystallite size (*D*_*c*_, S25I Fig for ASW and S28I Fig for FW). In ASW, *D*_*c*_ decreases from 64±1 Å at *t* = 0 d to 57.3±0.9 Å at *t* = 42 d, while in FW it remains nearly constant at approximately 62 Å. This suggests that microfibrils in ASW are more susceptible to UV-induced modifications than those in FW.

## Conclusions

MFs are emerging pollutants of great concern, and their study must go beyond toxicological assessments to include the physical and mechanical processes that influence their formation, transformation, and fate in the environment. This study examined the nanostructural evolution of five common MFs (cotton, linen, cellulose acetate, polyester, and polyamide) subjected to simulated solar UV irradiation equivalent to one year of environmental exposure, under both ASW and FW conditions. SAXS and WAXS were used to characterize their hierarchical structure, from microfibrils to macrofibrils. SAXS analysis, supported by a multiscale model of polydisperse core-shell microfibrils arranged in a distorted hexagonal lattice, revealed distinct material- and medium-dependent responses. In ASW, cotton and polyamide exhibited an increase in microfibril spacing, leading to significant macrofibril enlargement. In FW, cotton maintained a more stable structure, while polyamide showed an initial enlargement followed by re-compaction at later times. Conversely, linen and polyester underwent a progressive reduction in macrofibril diameter in both media, suggesting a greater susceptibility to UV-induced fragmentation. Cellulose acetate showed remarkable stability in ASW but displayed shrinkage and partial reorganization in FW, indicating a medium-specific restructuring process. WAXS confirmed the presence of crystalline domains in cotton, linen, and polyamide, and revealed medium-specific variations in crystallite size. In particular, ASW exposure resulted in measurable increases in crystallite size for cotton and polyamide, possibly reflecting enhanced crystallinity or chain packing during UV-induced reorganization. These structural changes may be linked to radical reactions initiated by UV exposure. UV radiation can generate free radicals within polymer double bonds, which, in the presence of dissolved molecular oxygen, trigger degradation pathways. The presence of salts in water can further modulate these effects, as the ions may alter radical activity along polymer chains. This may explain the observed differences between MFs degradation in FW and ASW. While this study focused on nanostructural evolution, it is important to recognize that degradation also involves chemical transformations, such as oxidation, hydrolysis, or chain scission [9]. These processes, though not directly investigated here, are often closely coupled with structural changes and may contribute to the observed rearrangements. A more comprehensive understanding would benefit from complementary chemical analyses aimed at elucidating the interplay between chemical and structural degradation pathways. The overall mechanisms of degradation observed in this work for MFs allow to suggest a possible link with their environmental fate and occurrence, also providing a possible explanation on what we observe in environmental samples, where the occurrence of natural polymers largely predominate. Notably, this study revealed a potentially reliable indicator for weathering of cotton fibers which exhibited the more marked increase of macrofibril diameter *D* because of solar energy and water permeation. Whether such nanostructural changes influence the environmental persistence of natural fibers remains an intriguing working hypothesis, requiring further experimental validation under realistic environmental conditions.

##  Supporting information

S1 File S1 Table. List of abbreviations used in the manuscript.S2 Table. Fiber types investigated in this study.S3 Table. Physical parameters considered for the evaluation of the fibers exposure time under the solar simulator apparatus.S4 Table. Correlation matrix of SAXS fitting parameters of cotton in ASW at *t* = 0 d.S5 Table. Correlation matrix of SAXS fitting parameters of cotton in FW at *t* = 0 d.S6 Table. Correlation matrix of SAXS fitting parameters of cellulose acetate in ASW at *t* = 0 d.S7 Table. Correlation matrix of SAXS fitting parameters of cellulose acetate in FW at *t* = 0 d.S8 Table. Correlation matrix of SAXS fitting parameters of polyamide in ASW at *t* = 0 d.S9 Table. Correlation matrix of SAXS fitting parameters of polyamide in FW at *t* = 0 d.S10 Table. Correlation matrix of SAXS fitting parameters of polyester in ASW at *t* = 0 d.S11 Table. Correlation matrix of SAXS fitting parameters of polyester in FW at *t* = 0 d.S12 Table. Correlation matrix of SAXS fitting parameters of linen in ASW at *t* = 0 d.S13 Table. Correlation matrix of SAXS fitting parameters of linen in FW at *t* = 0 d.S1 Fig. Modified caps of the bottles. S2 Fig. Comparison between glass and quartz transmittance.S3 Fig. Solar simulator used during the experiment.S4 Fig. SAXS cell used during the SAXS/WAXS experiment at Elettra.S5 Fig. Representation of the average MF cross section for MFs of cotton in ASW.S6 Fig. SAXS and WAXS curves of cotton MFs in FW.S7 Fig. Fitting parameters from SAXS analysis of MFs of cotton in FW.S8 Fig. Fitting parameters from WAXS analysis of MFs of cotton in FW.S9 Fig. SAXS and WAXS curves of cellulose acetate MFs in ASW.S10 Fig. Fitting parameters from SAXS analysis of MFs of cellulose acetate in ASW.S11 Fig. SAXS and WAXS curves of cellulose acetate MFs in FW.S12 Fig. Fitting parameters from SAXS analysis of MFs of cellulose acetate in FW.S13 Fig. SAXS and WAXS curves of polyamide MFs in ASW.S14 Fig. Fitting parameters from SAXS analysis of MFs of polyamide in ASW.S15 Fig. Fitting parameters from WAXS analysis of MFs of polyamide in ASW.S16 Fig. SAXS and WAXS curves of polyamide MFs in FW.S17 Fig. Fitting parameters from SAXS analysis of MFs of polyamide in FW.S18 Fig. Fitting parameters from WAXS analysis of MFs of polyamide in FW.S19 Fig. SAXS and WAXS curves of polyester MFs in ASW.S20 Fig. Fitting parameters from SAXS analysis of MFs of polyester in ASW.S21 Fig. SAXS and WAXS curves of polyester MFs in FW.S22 Fig. Fitting parameters from SAXS analysis of MFs of polyester in FW.S23 Fig. SAXS and WAXS curves of linen MFs in ASW.S24 Fig. Fitting parameters from SAXS analysis of MFs of linen in ASW.S25 Fig. Fitting parameters from WAXS analysis of MFs of linen in ASW.S26 Fig. SAXS and WAXS curves of linen MFs in FW.S27 Fig. Fitting parameters from SAXS analysis of MFs of linen in FW.S28 Fig. Fitting parameters from WAXS analysis of MFs of linen in FW.S29 Fig. Core-shell cross sections of microfibrils during UV irradiation.S30 Fig. Core radius probability distribution function.
(PDF)
