# Supplementary material for: Structural evolution of microfibers in seawater and freshwater under simulated sunlight: A small- and wide-angle X-ray scattering study
Source: PLoS One. 2025 Sep 10;20(9):e0328502. doi: 10.1371/journal.pone.0328502 (PMC12422520; doi:10.1371/journal.pone.0328502)
Supplement: S1 File — S1 Table. List of abbreviations used in the manuscript.S2 Table. Fiber types investigated in this study.S3 Table. Physical parameters considered for the evaluation of the fibers exposure time under the solar simulator apparatus.S4 Table. Correlation matrix of SAXS fitting parameters of cotton in ASW at t = 0 d.S5 Table. Correlation matrix of SAXS fitting parameters of cotton in FW at t = 0 d.S6 Table. Correlation matrix of SAXS fitting parameters of cellulose acetate in ASW at t = 0 d.S7 Table. Correlation matrix of SAXS fitting parameters of cellulose acetate in FW at t = 0 d.S8 Table. Correlation matrix of SAXS fitting parameters of polyamide in ASW at t = 0 d.S9 Table. Correlation matrix of SAXS fitting parameters of polyamide in FW at t = 0 d.S10 Table. Correlation matrix of SAXS fitting parameters of polyester in ASW at t = 0 d.S11 Table. Correlation matrix of SAXS fitting parameters of polyester in FW at t = 0 d.S12 Table. Correlation matrix of SAXS fitting parameters of linen in ASW at t = 0 d.S13 Table. Correlation matrix of SAXS fitting parameters of linen in FW at t = 0 d.S1 Fig. Modified caps of the bottles. S2 Fig. Comparison between glass and quartz transmittance.S3 Fig. Solar simulator used during the experiment.S4 Fig. SAXS cell used during the SAXS/WAXS experiment at Elettra.S5 Fig. Representation of the average MF cross section for MFs of cotton in ASW.S6 Fig. SAXS and WAXS curves of cotton MFs in FW.S7 Fig. Fitting parameters from SAXS analysis of MFs of cotton in FW.S8 Fig. Fitting parameters from WAXS analysis of MFs of cotton in FW.S9 Fig. SAXS and WAXS curves of cellulose acetate MFs in ASW.S10 Fig. Fitting parameters from SAXS analysis of MFs of cellulose acetate in ASW.S11 Fig. SAXS and WAXS curves of cellulose acetate MFs in FW.S12 Fig. Fitting parameters from SAXS analysis of MFs of cellulose acetate in FW.S13 Fig. SAXS and WAXS curves of polyamide MFs in ASW.S14 Fig. Fitting parameters from SAXS analysis of MFs of polyamide in ASW.S15 Fig. [file pone.0328502.s001.pdf]

Supporting information

Structural evolution of microfibers in seawater and freshwater under simulated sunlight: a Small- and Wide-Angle X-ray Scattering Study

Astra Piccinini[1], Giulia Lucia[1], Daniele Colarossi[2], Paolo Principi[2], Heinz Amenitsch[3], Lucia Pittura[1], Francesco Regoli[1], Stefania Gorbi[1] and Francesco Spinozzi[1]

[1] Department of Life and Environmental Sciences, Polytechnic University of Marche, Ancona, 60131, Italy

[2] Department of Industrial Engineering and Mathematical Sciences, Polytechnic University of Marche, Ancona, 60131, Italy

[3] Institute for Inorganic Chemistry, Graz University of Technology, Graz, 8010, Austria

**Abbreviation**	**Definition**
ASW	Artificial Seawater
FW	Freshwater
MF	Microfiber
SAXS	Small-Angle X-ray Scattering
SAXSDOG	SAXS Data Organization and Graphics (software)
WAXS	Wide-Angle X-ray Scattering
SANS	Small-Angle Neutron Scattering
UV	Ultraviolet
FTIR	Fourier Transform Infrared Spectroscopy
μFTIR	Micro Fourier Transform Infrared Spectroscopy
SEM	Scanning Electron Microscopy
XPS	X-ray Photoelectron Spectroscopy
TGA	Thermogravimetric Analysis
GSAS	General Structure Analysis System
IPCB	Institute of Physical Chemistry of the CNR
CNR	National Research Council (Italy)
CIF	Crystallographic Information File
EXPGUI	Graphical User Interface for GSAS

S1 Table: List of abbreviations used in the manuscript.

**Fiber Type**
Cotton
Cellulose Acetate
Polyamide
Polyester
Linen

S2 Table: Fiber types investigated in this study.

Mean solar irradiance (kWh/m^2)	1200
5% UV component (kWh/m^2)	60
UV component of solar simulator power (kW/m^2)	0.06
Exposition time per day (h)	24
Total energy per day (kWh/m^2)	1.44
Total exposition time to simulate 1 year of solar exposure (days)	41.67

S3 Table: **Physical parameters considered for the evaluation of the fibers exposure time under the solar simulator apparatus**.

	$<R>$	a	g_a	N_a	g_R	ϕ	δ
$<R>$	1.000						
a	-0.005	1.000					
g_a	-0.004	-0.148	1.000				
N_a	0.000	0.000	0.000	1.000			
g_R	-0.005	0.127	-0.127	0.000	1.000		
ϕ	0.000	0.113	-0.013	0.000	-0.050	1.000	
δ	0.000	-0.005	0.000	0.000	0.000	0.000	1.000

S4 Table: **Correlation matrix of SAXS fitting parameters of cotton in ASW at $t = 0$ d**.

	$<R>$	a	g_a	N_a	g_R	ϕ	δ
$<R>$	1.000						
a	-0.260	1.000					
g_a	0.012	0.031	1.000				
N_a	0.000	0.000	0.000	1.000			
g_R	-0.080	0.273	-0.029	0.000	1.000		
ϕ	-0.051	0.084	0.000	0.000	-0.023	1.000	
δ	-0.079	-0.166	0.003	0.000	0.068	0.000	1.000

S5 Table: **Correlation matrix of SAXS fitting parameters of cotton in FW at $t = 0$ d**.

	$<R>$	a	g_a	N_a	g_R	ϕ	δ
$<R>$	1.000						
a	-0.076	1.000					
g_a	-0.142	-0.123	1.000				
N_a	0.000	0.000	0.000	1.000			
g_R	-0.230	0.101	0.035	0.000	1.000		
ϕ	-0.015	0.050	0.009	0.000	-0.051	1.000	
δ	-0.057	-0.202	-0.044	0.000	0.016	-0.075	1.000

S6 Table: **Correlation matrix of SAXS fitting parameters of cellulose acetate in ASW at $t = 0$ d**.

	$<R>$	a	g_a	N_a	g_R	ϕ	δ
$<R>$	1.000						
a	0.000	1.000					
g_a	0.000	0.000	1.000				
N_a	-0.168	0.000	0.000	1.000			
g_R	0.000	0.000	0.000	0.000	1.000		
ϕ	0.000	0.000	0.016	0.000	0.049	1.000	
δ	0.000	0.000	-0.012	0.000	-0.033	0.086	1.000

S7 Table: Correlation matrix of SAXS fitting parameters of cellulose acetate in FW at $t = 0$ d.

	$<R>$	a	g_a	N_a	g_R	ϕ	δ	b
$<R>$	1.000							
a	-0.185	1.000						
g_a	0.135	-0.090	1.000					
N_a	0.000	0.000	0.000	1.000				
g_R	-0.220	0.167	-0.192	0.000	1.000			
ϕ	-0.005	0.000	0.003	0.000	0.000	1.000		
δ	-0.115	-0.290	-0.061	0.000	0.026	0.000	1.000	
b	0.006	-0.298	0.060	0.000	0.048	0.006	-0.124	1.000

S8 Table: Correlation matrix of SAXS fitting parameters of polyamide in ASW at $t = 0$ d.

	$<R>$	a	g_a	N_a	g_R	ϕ	δ	b
$<R>$	1.000							
a	0.000	1.000						
g_a	0.003	0.000	1.000					
N_a	0.000	0.000	0.000	1.000				
g_R	-0.110	0.003	0.004	0.000	1.000			
ϕ	-0.020	0.000	0.004	0.000	-0.029	1.000		
δ	-0.058	0.000	0.003	0.000	0.008	-0.003	1.000	
b	0.217	-0.007	-0.277	0.000	0.276	0.092	0.030	1.000

S9 Table: Correlation matrix of SAXS fitting parameters of polyamide in FW at $t = 0$ d.

	$<R>$	a	g_a	N_a	g_R	ϕ	δ
$<R>$	1.000						
a	0.000	1.000					
g_a	0.000	0.000	1.000				
N_a	0.000	0.000	0.000	1.000			
g_R	0.000	0.000	0.000	0.000	1.000		
ϕ	0.000	0.000	0.000	0.000	0.000	1.000	
δ	0.000	0.000	0.000	0.000	0.000	0.000	1.000

S10 Table: Correlation matrix of SAXS fitting parameters of polyester in ASW at $t = 0$ d.

	$<R>$	a	g_a	N_a	g_R	ϕ	δ
$<R>$	1.000						
a	-0.136	1.000					
g_a	-0.231	0.072	1.000				
N_a	0.000	0.000	0.000	1.000			
g_R	-0.236	0.202	0.212	0.000	1.000		
ϕ	0.000	0.031	-0.021	0.000	-0.058	1.000	
δ	-0.161	-0.197	-0.188	0.000	0.018	-0.197	1.000

S11 Table: Correlation matrix of SAXS fitting parameters of polyester in FW at $t = 0$ d.

	$<R>$	a	g_a	N_a	g_R	ϕ	δ
$<R>$	1.000						
a	-0.115	1.000					
g_a	-0.004	-0.010	1.000				
N_a	0.000	0.000	0.000	1.000			
g_R	-0.115	0.059	0.003	0.000	1.000		
ϕ	-0.005	0.009	0.000	0.000	0.000	1.000	
δ	-0.101	-0.283	0.000	0.000	0.106	-0.003	1.000

S12 Table: Correlation matrix of SAXS fitting parameters of linen in ASW at $t = 0$ d.

	$<R>$	a	g_a	N_a	g_R	ϕ	δ
$<R>$	1.000						
a	-0.134	1.000					
g_a	-0.013	-0.024	1.000				
N_a	0.000	0.000	0.000	1.000			
g_R	-0.134	0.126	0.013	0.000	1.000		
ϕ	-0.038	0.104	-0.002	0.000	-0.059	1.000	
δ	-0.179	-0.128	-0.007	0.000	0.160	-0.045	1.000

S13 Table: Correlation matrix of SAXS fitting parameters of linen in FW at $t = 0$ d.

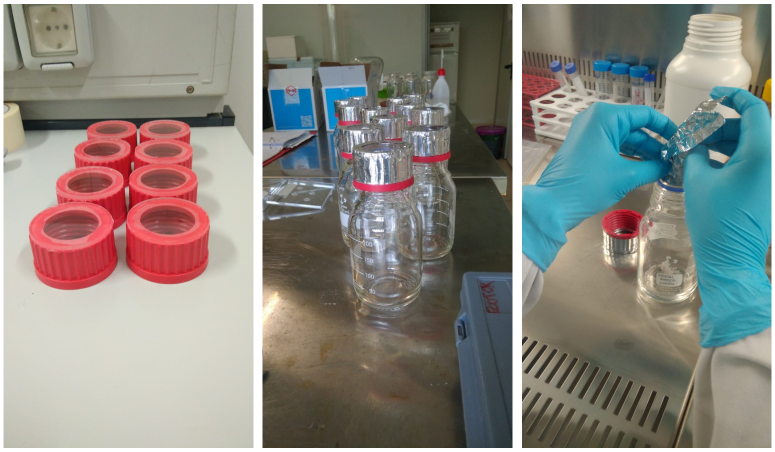

S1 Fig: Modified caps of the bottles. It is shown the top surface made of quartz (left) and the bottles ready to be filled with fibers (center and right).

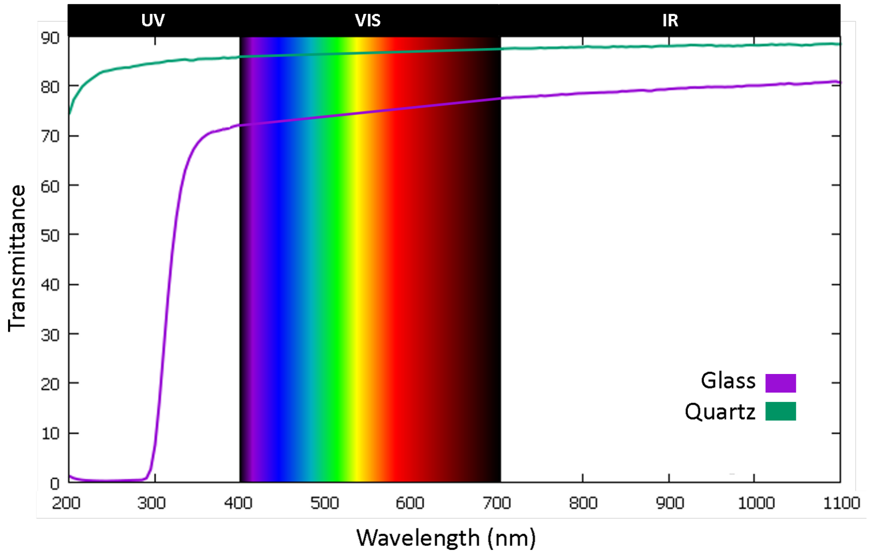

S2 Fig: Comparison between glass and quartz transmittance. The spectrum of the solar radiation has been considered.

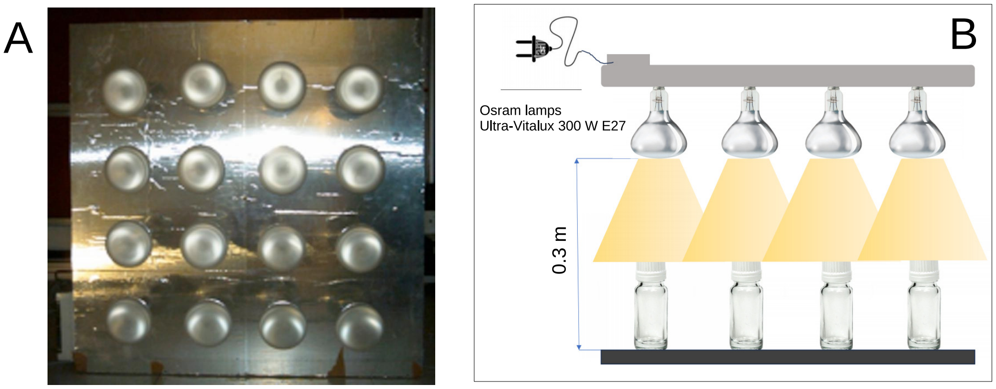

S3 Fig: Solar simulator used during the experiment.

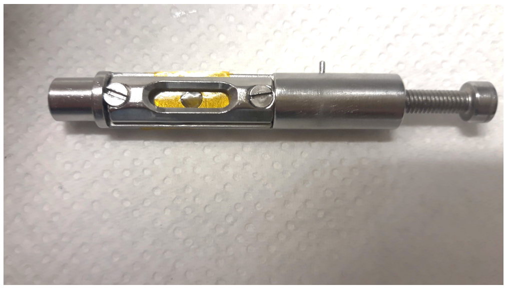

S4 Fig: SAXS cell used during the SAXS/WAXS experiment at Elettra. The solid sample of the fibers was adjusted inside the cell and then irradiated by X-ray.

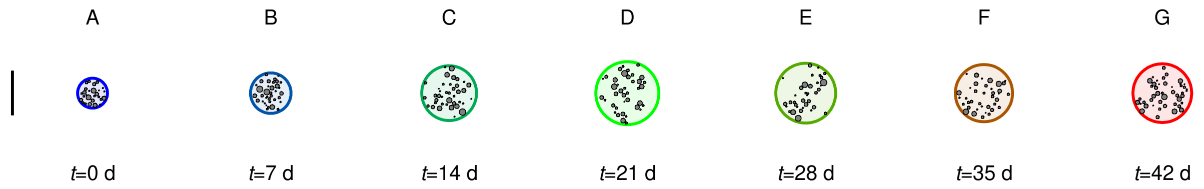

S5 Fig: **Representation of the average MF cross section for MFs of cotton in ASW**. The small gray circles, representing the nanofibers, have been drawn with a radius sampled by the fitted Gaussian poly-dispersed distribution and in a position sampled according to the bidimensional para-crystal fitting parameters a, g_a and N_a. The vertical black line on the left of panel A represents the length of 1000 Å.

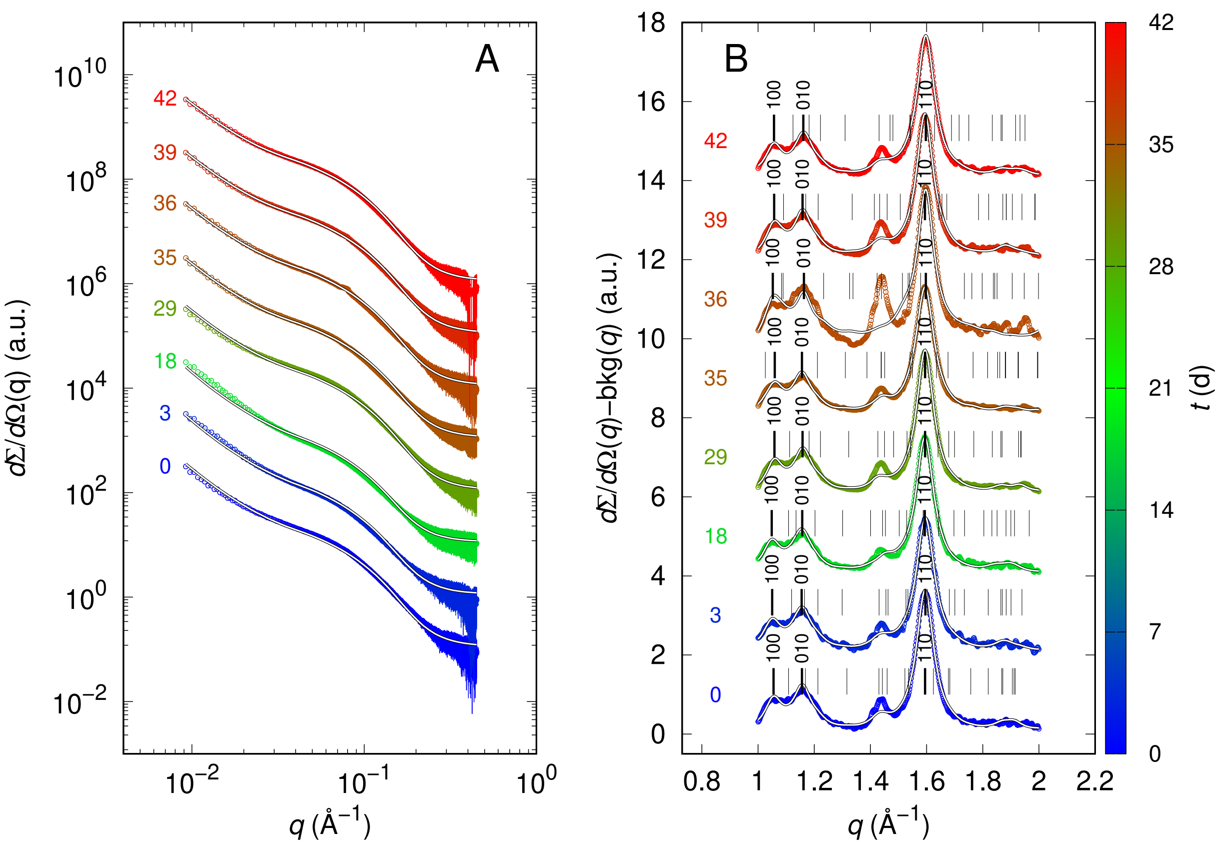

S6 Fig: **SAXS and WAXS curves of cotton MFs in FW**. The color of the data points corresponds to the vertical color scale, which indicates the UV irradiation time. A) The solid black and white lines represent the best fits obtained using GENFIT. Starting from the bottom, each curve is multiplied by a factor of 10 relative to the one below it, for clarity. B) The black and white lines correspond to the best fits obtained using GSAS. The experimental curves (shown without error bars for clarity) and their respective fits are presented, with the diffuse background, determined from the fit, subtracted from each. Thin vertical black lines indicate the Bragg reflections of the space group. Thick vertical black lines, with the corresponding Miller indices indicated, mark the positions of the main peaks. The curves are vertically offset by a factor of 2 for clarity.

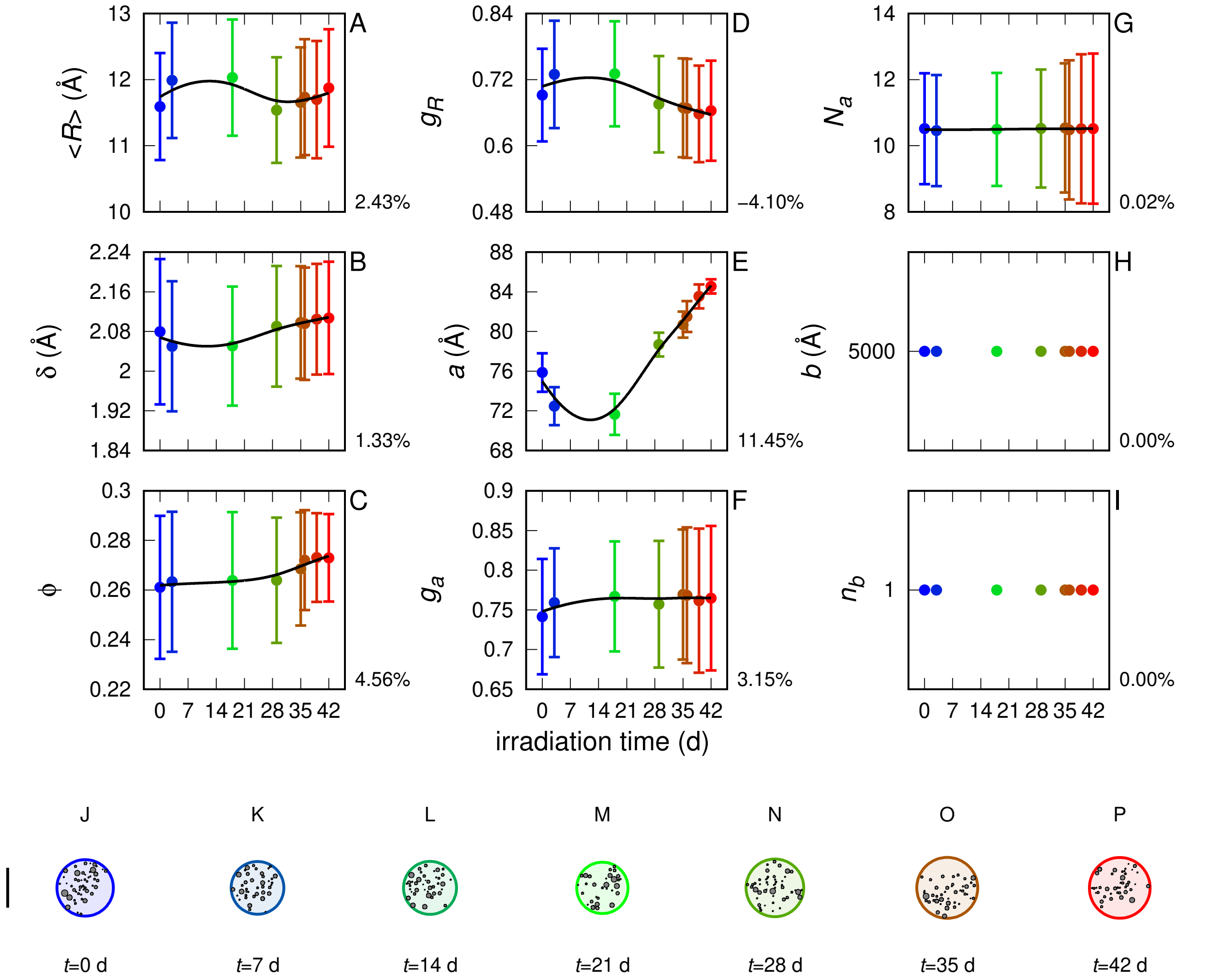

S7 Fig: **Fitting parameters from SAXS analysis of MFs of cotton in FW**. Smooth black curves among the points have been obtained with cubic splines weighted with uncertainties of the parameters. In the lower right corner of each panel, the percentage ratio between the difference from the final value ($t = 42$ d) and the initial value of the parameter ($t = 0$ d), relative to the initial value, is shown. Panels J-P: representation of the average MF cross section according to the fitting parameters. The small gray circles, representing the nanofibers, have been drawn with a radius sampled by the fitted Gaussian poly-dispersed distribution and in a position sampled according to the bidimensional para-crystal fitting parameters a, g_a and N_a. The vertical black line on the left of panel J represents the length of 1000 Å.

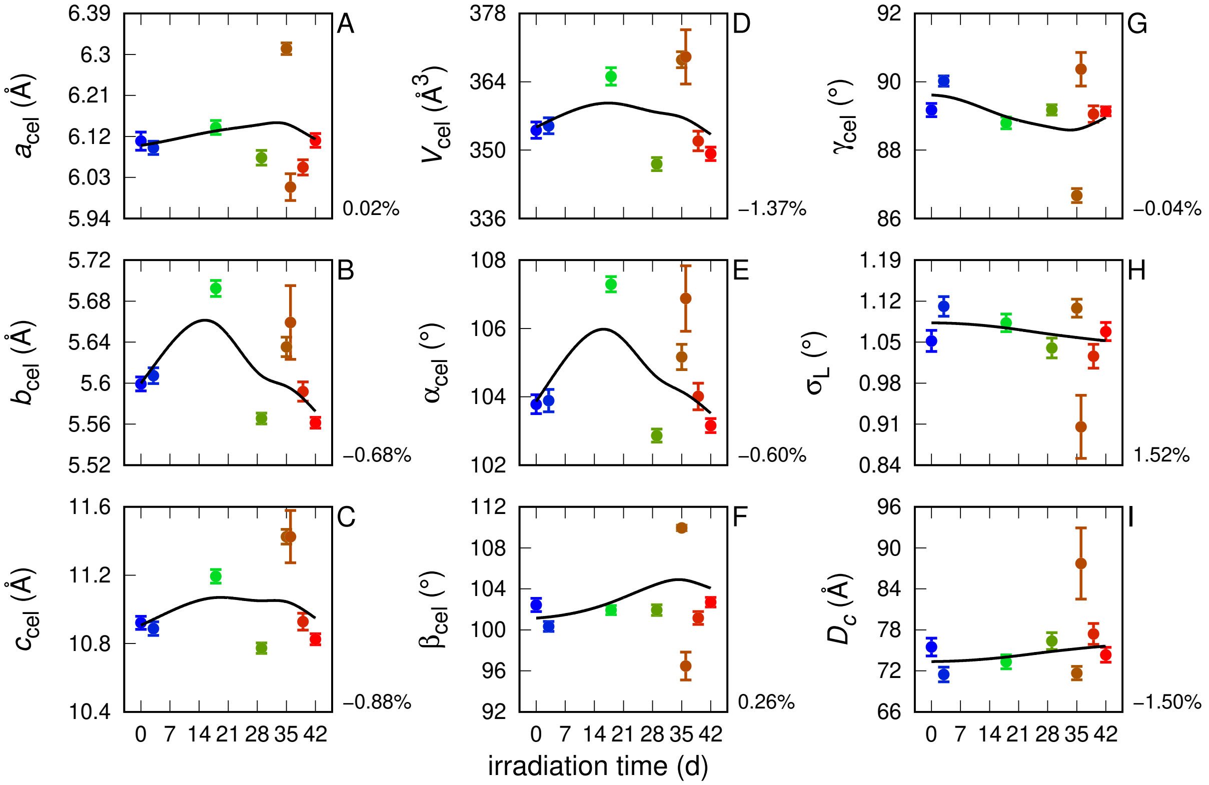

S8 Fig: **Fitting parameters from WAXS analysis of MFs of cotton in FW**. Smooth black curves among the points have been obtained with cubic splines weighted with uncertainties of the parameters. In the lower right corner of each panel, the percentage change between $t = 0$ d and $t = 42$ d, relative to the initial value, is reported.

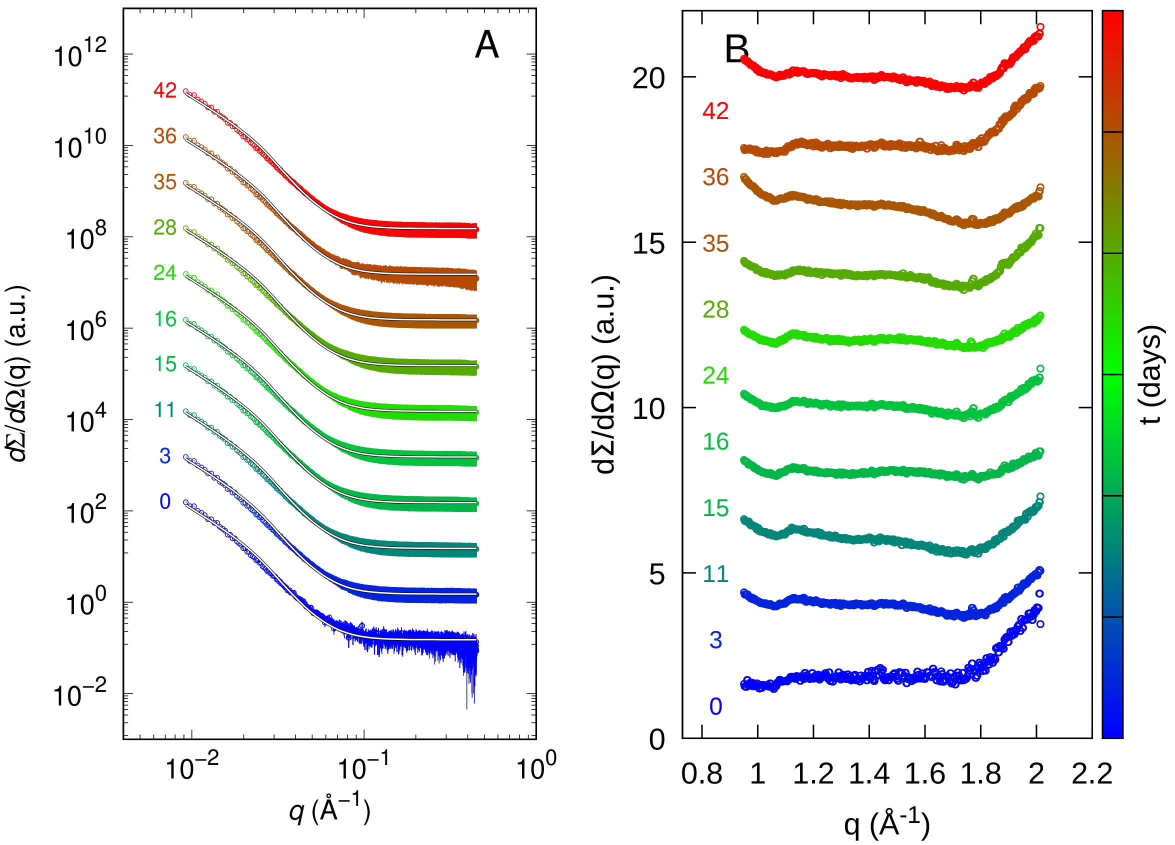

S9 Fig: **SAXS and WAXS curves of cellulose acetate MFs in ASW**. The color of the data points corresponds to the vertical color scale, which indicates the UV irradiation time. A) The solid black and white lines represent the best fits obtained using GENFIT. Starting from the bottom, each curve is multiplied by a factor of 10 relative to the one below it, for clarity. B) The black and white lines correspond to the best fits obtained using GSAS. The experimental curves (shown without error bars for clarity) and their respective fits are presented, with the diffuse background, determined from the fit, subtracted from each. Thin vertical black lines indicate the Bragg reflections of the space group. Thick vertical black lines, with the corresponding Miller indices indicated, mark the positions of the main peaks. The curves are vertically offset by a factor of 2 for clarity.

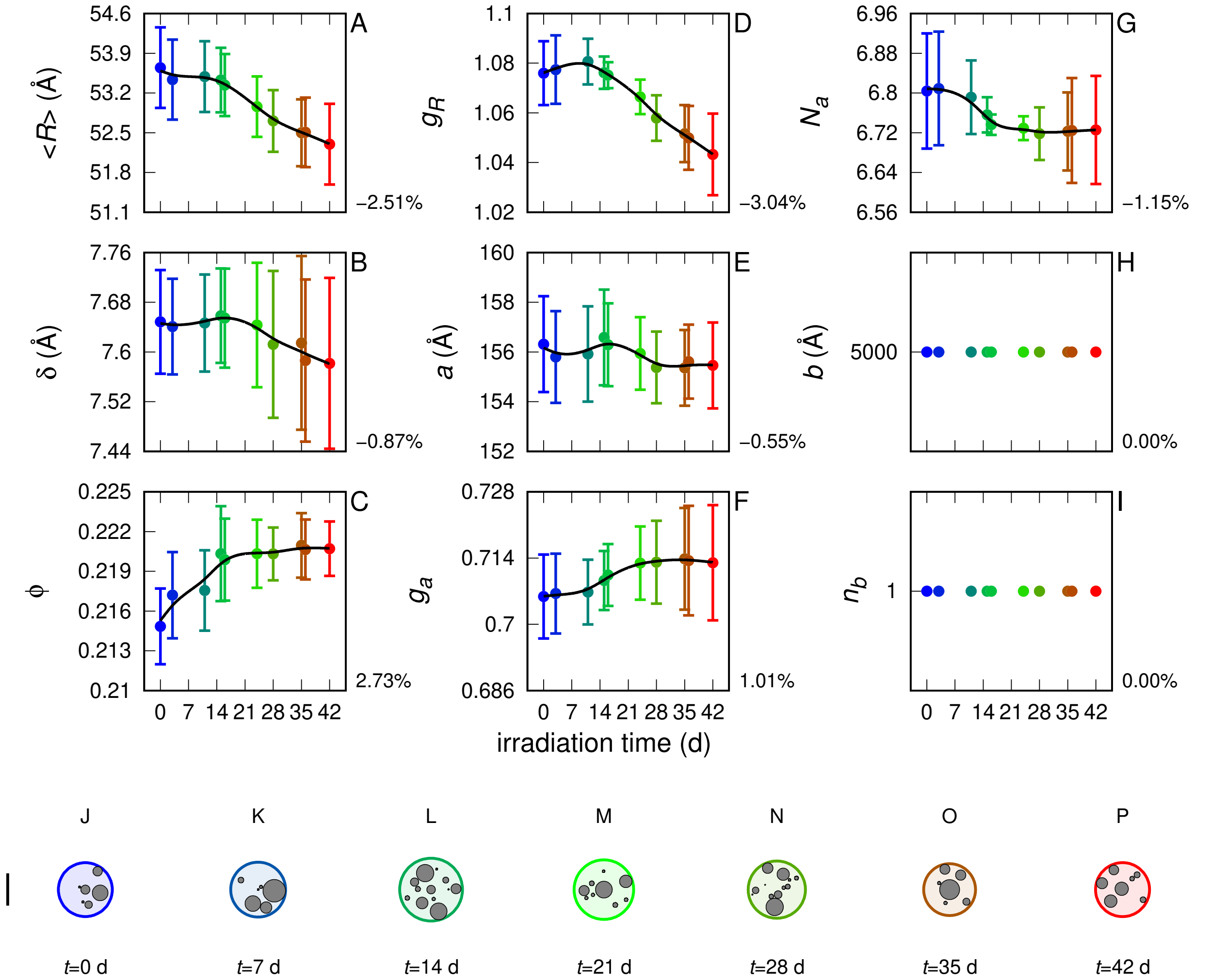

S10 Fig: **Fitting parameters from SAXS analysis of MFs of cellulose acetate in ASW**. Smooth black curves among the points have been obtained with cubic splines weighted with uncertainties of the parameters. In the lower right corner of each panel, the percentage ratio between the difference from the final value (t = 42 d) and the initial value of the parameter (t = 0 d), relative to the initial value, is shown. Panels J-P: representation of the average MF cross section according to the fitting parameters. The small gray circles, representing the nanofibers, have been drawn with a radius sampled by the fitted Gaussian poly-dispersed distribution and in a position sampled according to the bidimensional para-crystal fitting parameters a, g_a and N_a. The vertical black line on the left of panel J represents the length of 1000 Å.

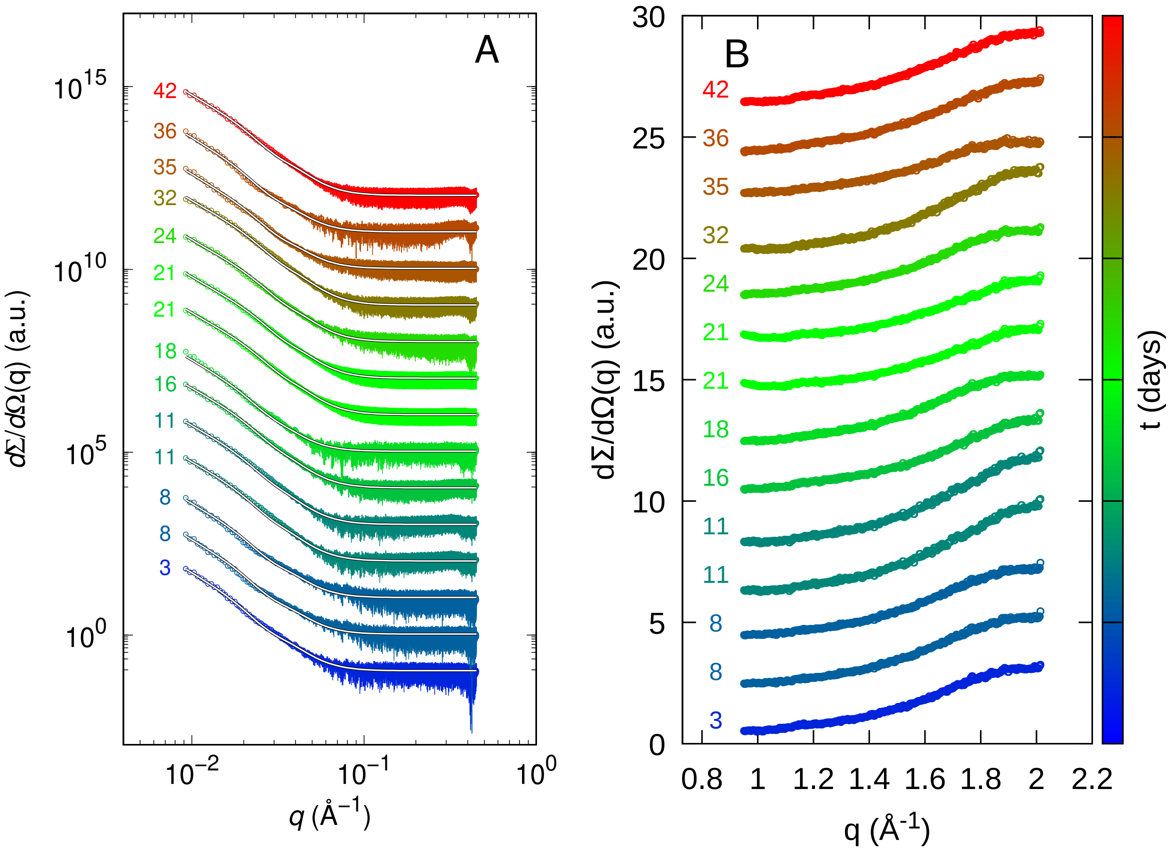

S11 Fig: **SAXS and WAXS curves of cellulose acetate MFs in FW**. The color of the data points corresponds to the vertical color scale, which indicates the UV irradiation time. A) The solid black and white lines represent the best fits obtained using GENFIT. Starting from the bottom, each curve is multiplied by a factor of 10 relative to the one below it, for clarity. B) The black and white lines correspond to the best fits obtained using GSAS. The experimental curves (shown without error bars for clarity) and their respective fits are presented, with the diffuse background, determined from the fit, subtracted from each. Thin vertical black lines indicate the Bragg reflections of the space group. Thick vertical black lines, with the corresponding Miller indices indicated, mark the positions of the main peaks. The curves are vertically offset by a factor of 2 for clarity.

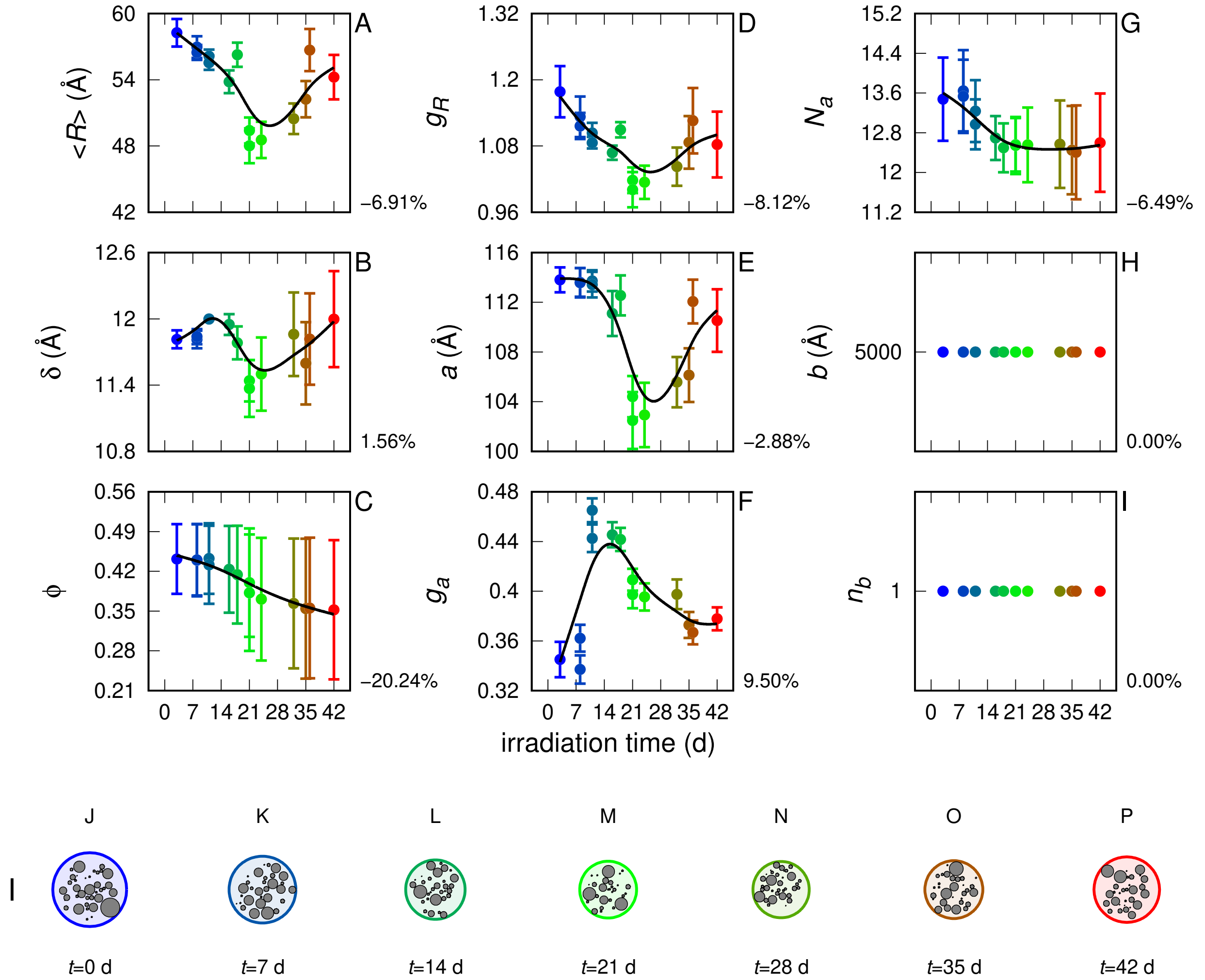

S12 Fig: **Fitting parameters from SAXS analysis of MFs of cellulose acetate in FW**. Smooth black curves among the points have been obtained with cubic splines weighted with uncertainties of the parameters. In the lower right corner of each panel, the percentage ratio between the difference from the final value ($t = 42$ d) and the initial value of the parameter ($t = 0$ d), relative to the initial value, is shown. Panels J-P: representation of the average MF cross section according to the fitting parameters. The small gray circles, representing the nanofibers, have been drawn with a radius sampled by the fitted Gaussian poly-dispersed distribution and in a position sampled according to the bidimensional para-crystal fitting parameters a, g_a and N_a. The vertical black line on the left of panel J represents the length of 1000 Å.

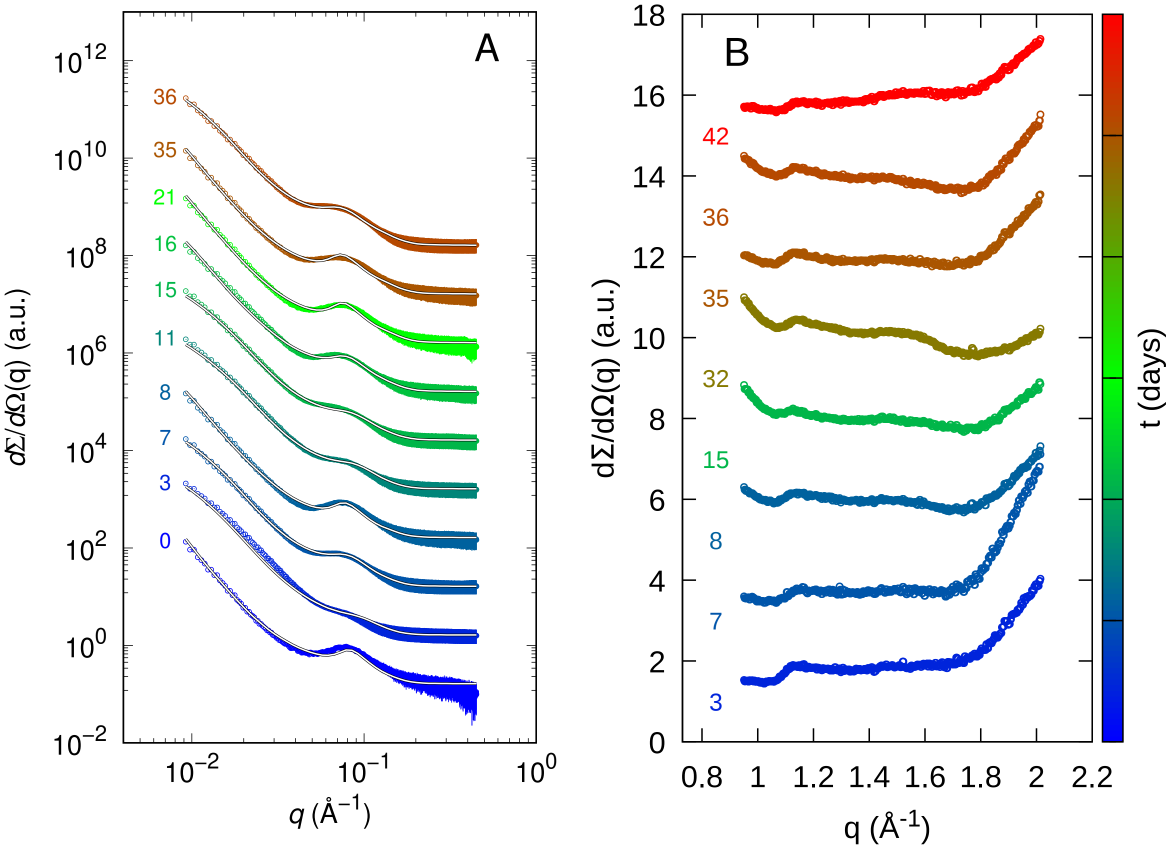

S13 Fig: **SAXS and WAXS curves of polyamide MFs in ASW**. The color of the data points corresponds to the vertical color scale, which indicates the UV irradiation time. A) The solid black and white lines represent the best fits obtained using GENFIT. Starting from the bottom, each curve is multiplied by a factor of 10 relative to the one below it, for clarity. B) The black and white lines correspond to the best fits obtained using GSAS. The experimental curves (shown without error bars for clarity) and their respective fits are presented, with the diffuse background, determined from the fit, subtracted from each. Thin vertical black lines indicate the Bragg reflections of the space group. Thick vertical black lines, with the corresponding Miller indices indicated, mark the positions of the main peaks. The curves are vertically offset by a factor of 2 for clarity.

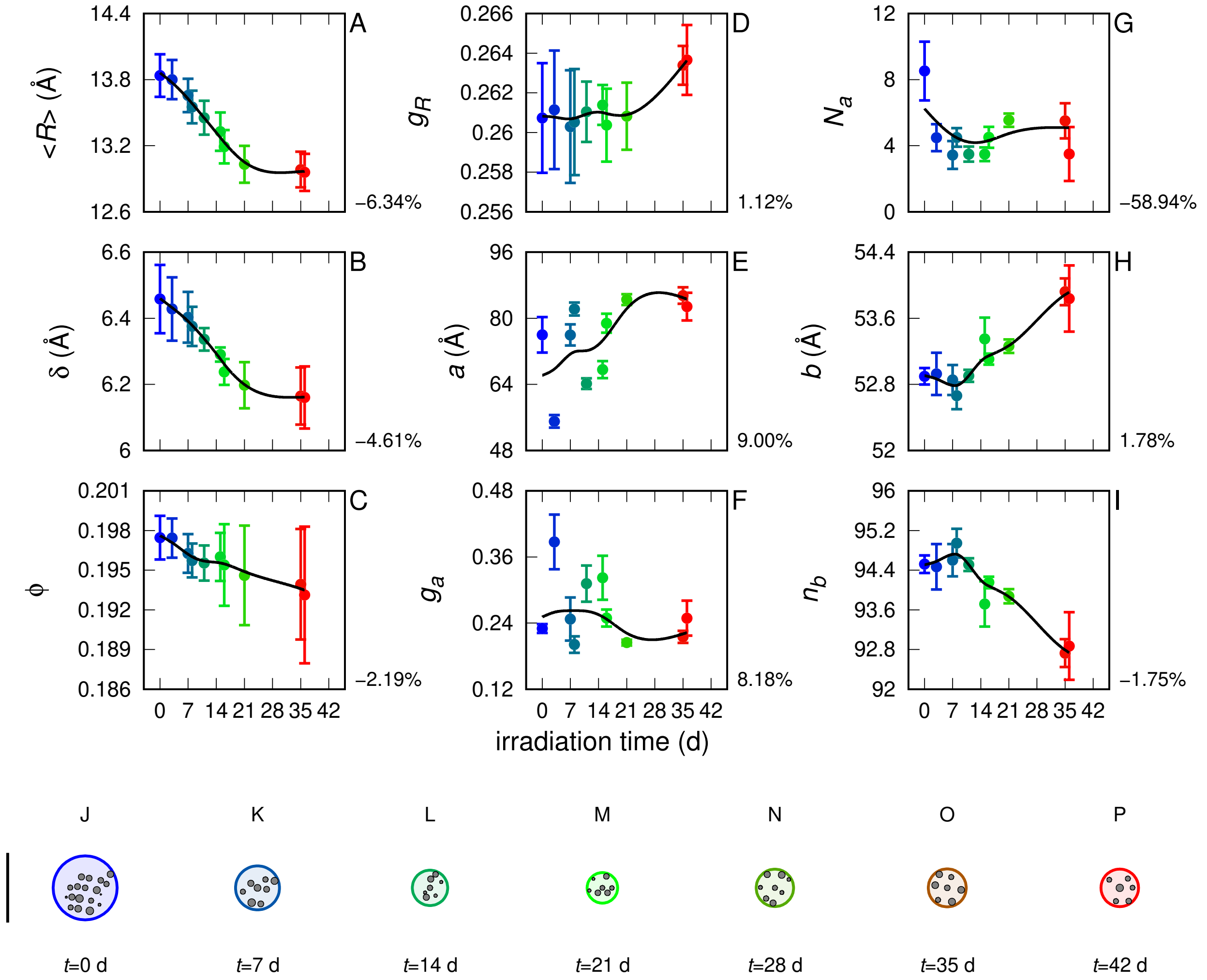

S14 Fig: **Fitting parameters from SAXS analysis of MFs of polyamide in ASW**. Smooth black curves among the points have been obtained with cubic splines weighted with uncertainties of the parameters. In the lower right corner of each panel, the percentage ratio between the difference from the final value ($t = 42$ d) and the initial value of the parameter ($t = 0$ d), relative to the initial value, is shown. Panels J-P: representation of the average MF cross section according to the fitting parameters. The small gray circles, representing the nanofibers, have been drawn with a radius sampled by the fitted Gaussian poly-dispersed distribution and in a position sampled according to the bidimensional para-crystal fitting parameters a, g_a and N_a. The vertical black line on the left of panel J represents the length of 1000 Å.

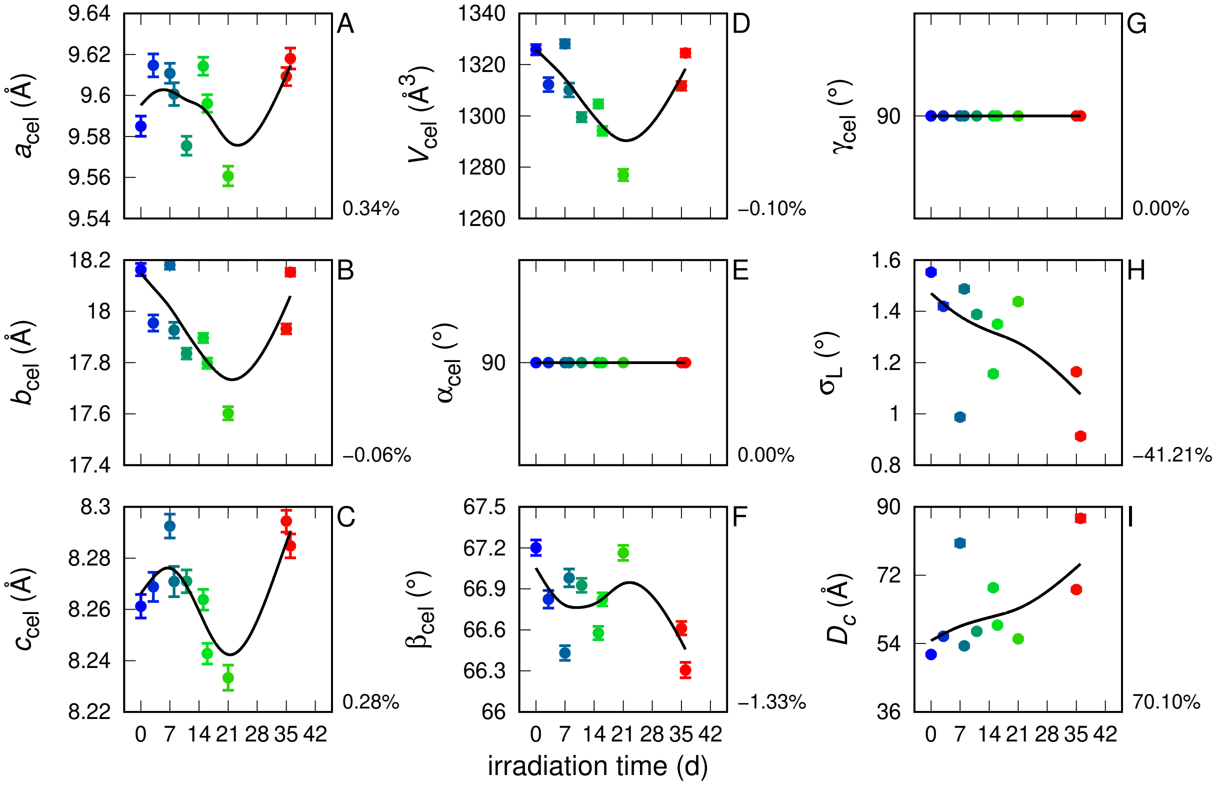

S15 Fig: **Fitting parameters from WAXS analysis of MFs of polyamide in ASW**. Smooth black curves among the points have been obtained with cubic splines weighted with uncertainties of the parameters. In the lower right corner of each panel, the percentage change between $t = 0$ d and $t = 42$ d, relative to the initial value, is reported.

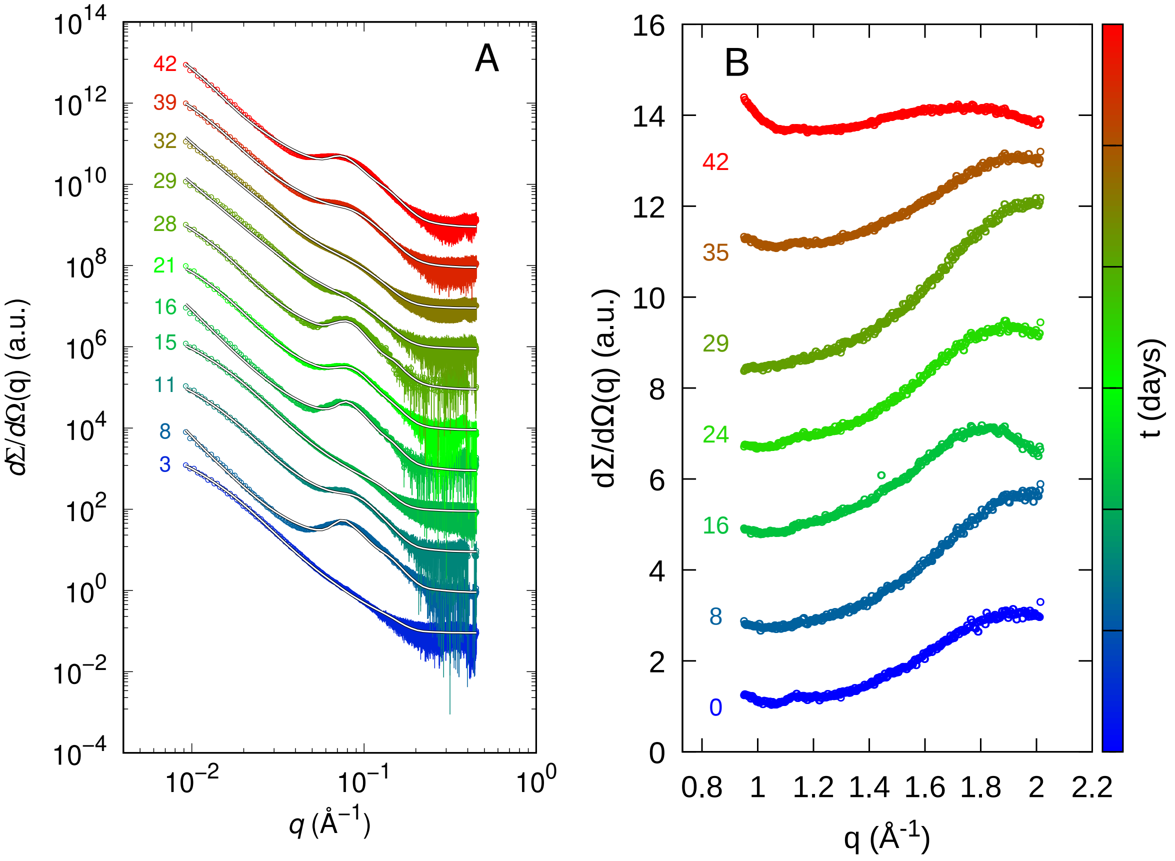

S16 Fig: **SAXS and WAXS curves of polyamide MFs in FW**. The color of the data points corresponds to the vertical color scale, which indicates the UV irradiation time. A) The solid black and white lines represent the best fits obtained using GENFIT. Starting from the bottom, each curve is multiplied by a factor of 10 relative to the one below it, for clarity. B) The black and white lines correspond to the best fits obtained using GSAS. The experimental curves (shown without error bars for clarity) and their respective fits are presented, with the diffuse background, determined from the fit, subtracted from each. Thin vertical black lines indicate the Bragg reflections of the space group. Thick vertical black lines, with the corresponding Miller indices indicated, mark the positions of the main peaks. The curves are vertically offset by a factor of 2 for clarity.

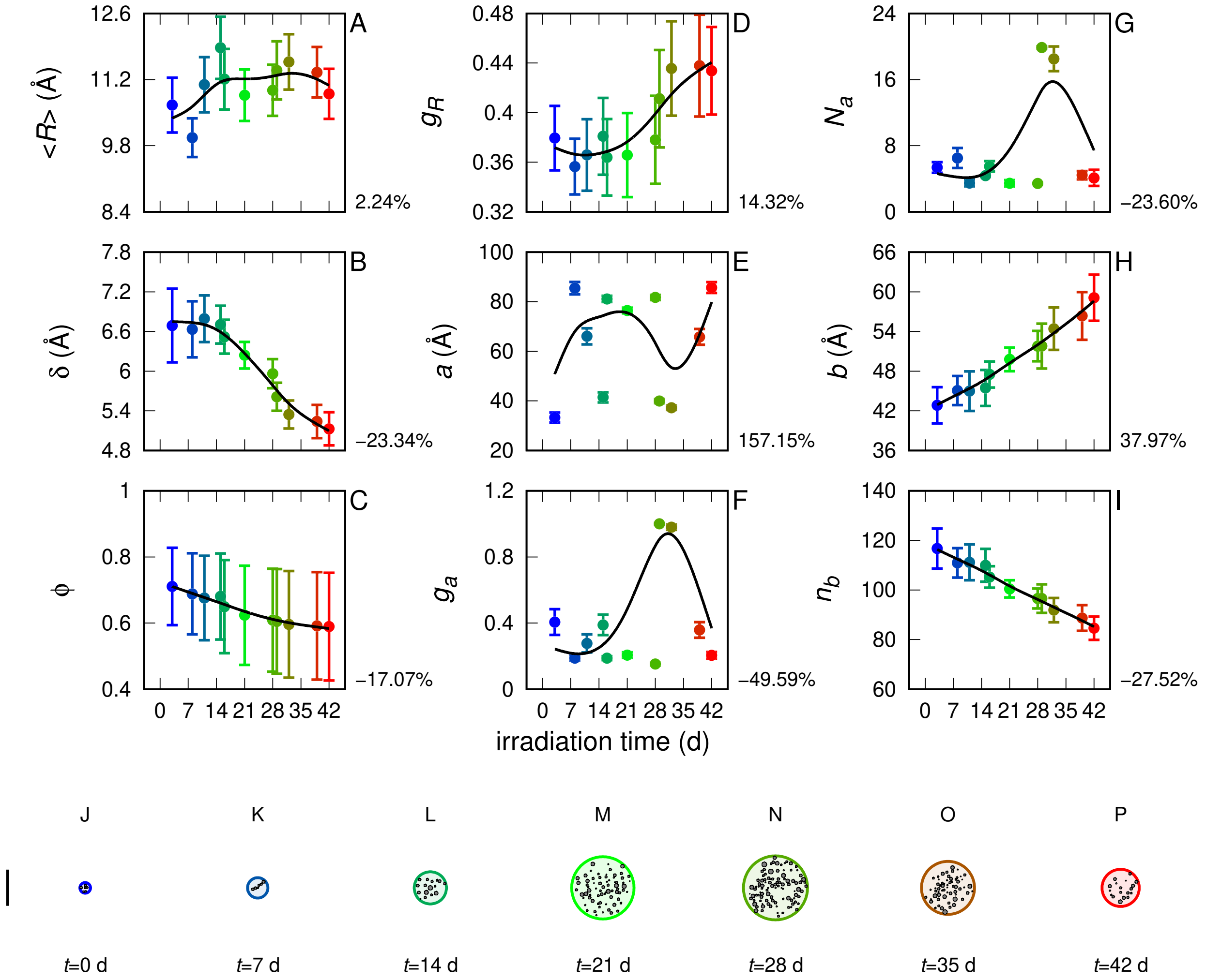

S17 Fig: **Fitting parameters from SAXS analysis of MFs of polyamide in FW**. Smooth black curves among the points have been obtained with cubic splines weighted with uncertainties of the parameters. In the lower right corner of each panel, the percentage ratio between the difference from the final value ($t = 42$ d) and the initial value of the parameter ($t = 0$ d), relative to the initial value, is shown. Panels J-P: representation of the average MF cross section according to the fitting parameters. The small gray circles, representing the nanofibers, have been drawn with a radius sampled by the fitted Gaussian poly-dispersed distribution and in a position sampled according to the bidimensional para-crystal fitting parameters a, g_a and N_a. The vertical black line on the left of panel J represents the length of 1000 Å.

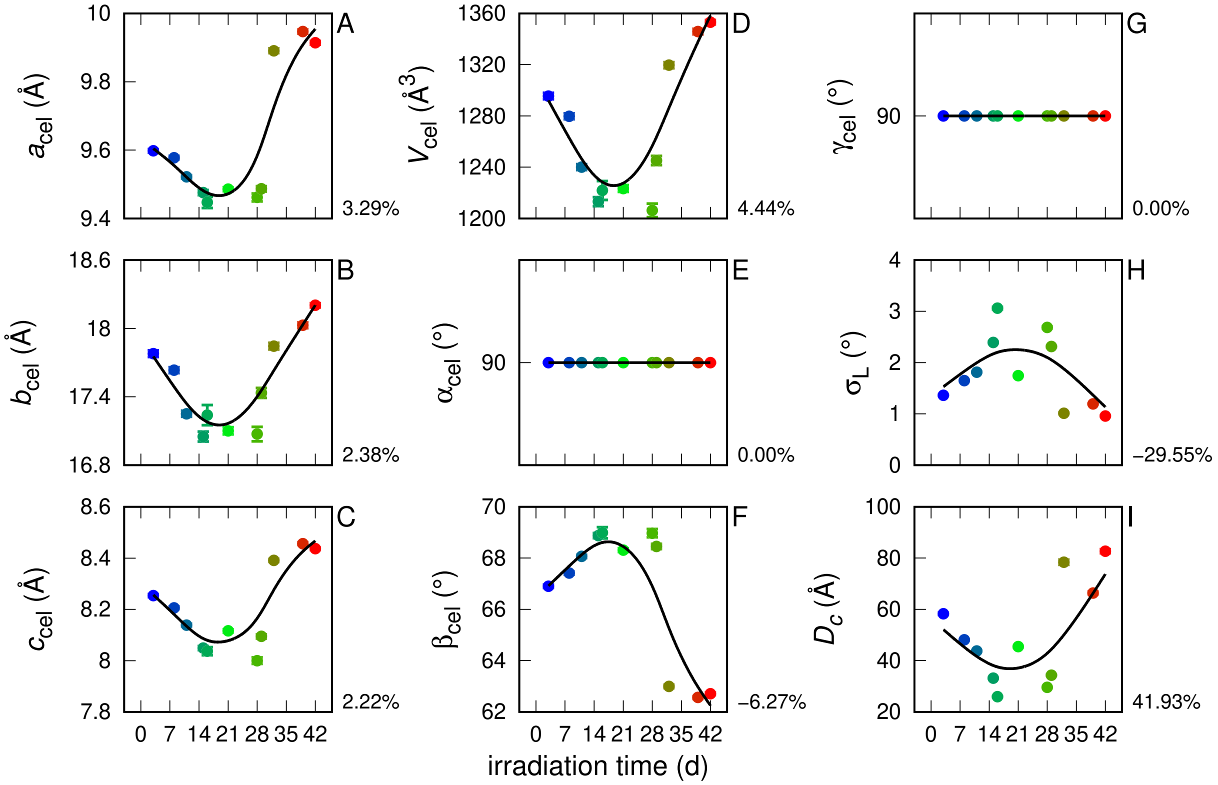

S18 Fig: **Fitting parameters from WAXS analysis of MFs of polyamide in FW**. Smooth black curves among the points have been obtained with cubic splines weighted with uncertainties of the parameters. In the lower right corner of each panel, the percentage change between $t = 0$ d and $t = 42$ d, relative to the initial value, is reported.

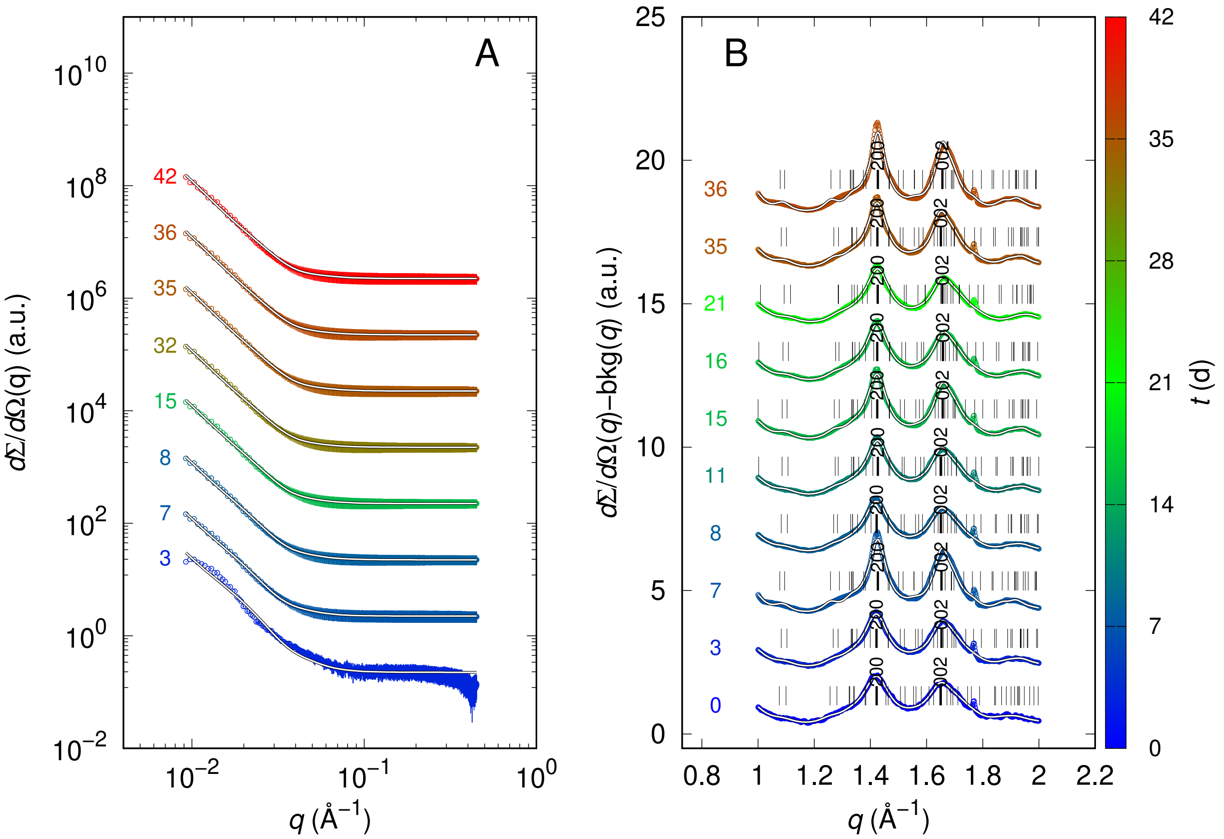

S19 Fig: **SAXS and WAXS curves of polyester MFs in ASW**. The color of the data points corresponds to the vertical color scale, which indicates the UV irradiation time. A) The solid black and white lines represent the best fits obtained using GENFIT. Starting from the bottom, each curve is multiplied by a factor of 10 relative to the one below it, for clarity. B) The black and white lines correspond to the best fits obtained using GSAS. The experimental curves (shown without error bars for clarity) and their respective fits are presented, with the diffuse background, determined from the fit, subtracted from each. Thin vertical black lines indicate the Bragg reflections of the space group. Thick vertical black lines, with the corresponding Miller indices indicated, mark the positions of the main peaks. The curves are vertically offset by a factor of 2 for clarity.

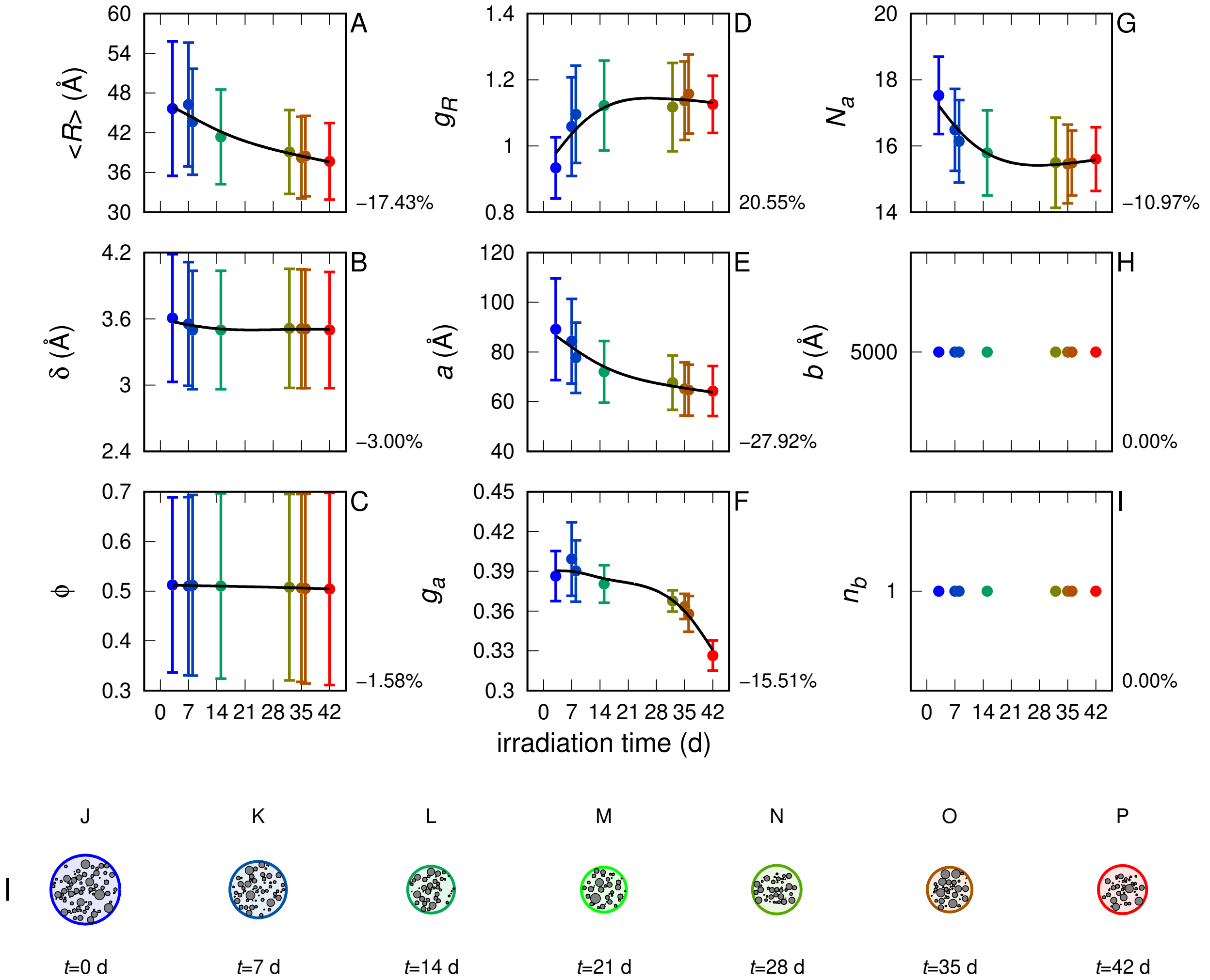

S20 Fig: **Fitting parameters from SAXS analysis of MFs of polyester in ASW**. Smooth black curves among the points have been obtained with cubic splines weighted with uncertainties of the parameters. In the lower right corner of each panel, the percentage ratio between the difference from the final value ($t = 42$ d) and the initial value of the parameter ($t = 0$ d), relative to the initial value, is shown. Panels J-P: representation of the average MF cross section according to the fitting parameters. The small gray circles, representing the nanofibers, have been drawn with a radius sampled by the fitted Gaussian poly-dispersed distribution and in a position sampled according to the bidimensional para-crystal fitting parameters a, g_a and N_a. The vertical black line on the left of panel J represents the length of 1000 Å.

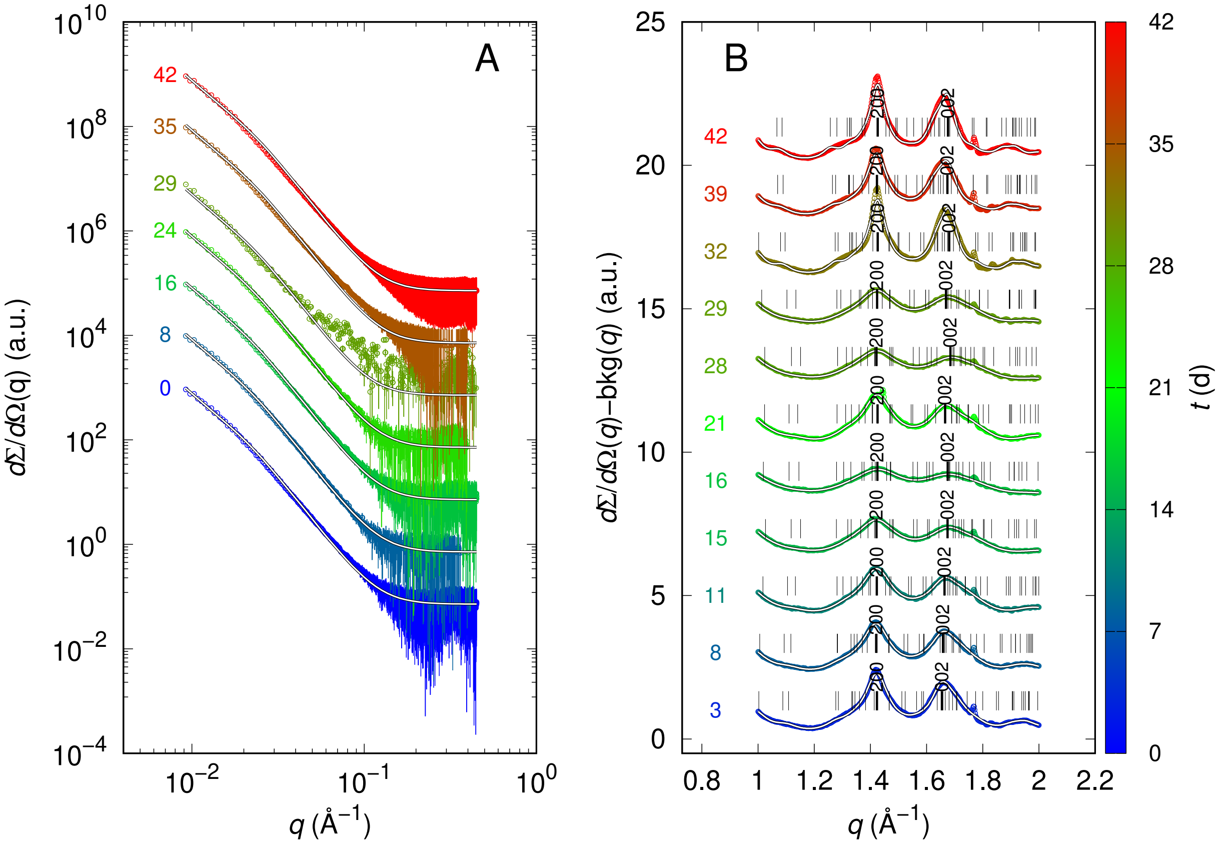

S21 Fig: **SAXS and WAXS curves of polyester MFs in FW**. The color of the data points corresponds to the vertical color scale, which indicates the UV irradiation time. A) The solid black and white lines represent the best fits obtained using GENFIT. Starting from the bottom, each curve is multiplied by a factor of 10 relative to the one below it, for clarity. B) The black and white lines correspond to the best fits obtained using GSAS. The experimental curves (shown without error bars for clarity) and their respective fits are presented, with the diffuse background, determined from the fit, subtracted from each. Thin vertical black lines indicate the Bragg reflections of the space group. Thick vertical black lines, with the corresponding Miller indices indicated, mark the positions of the main peaks. The curves are vertically offset by a factor of 2 for clarity.

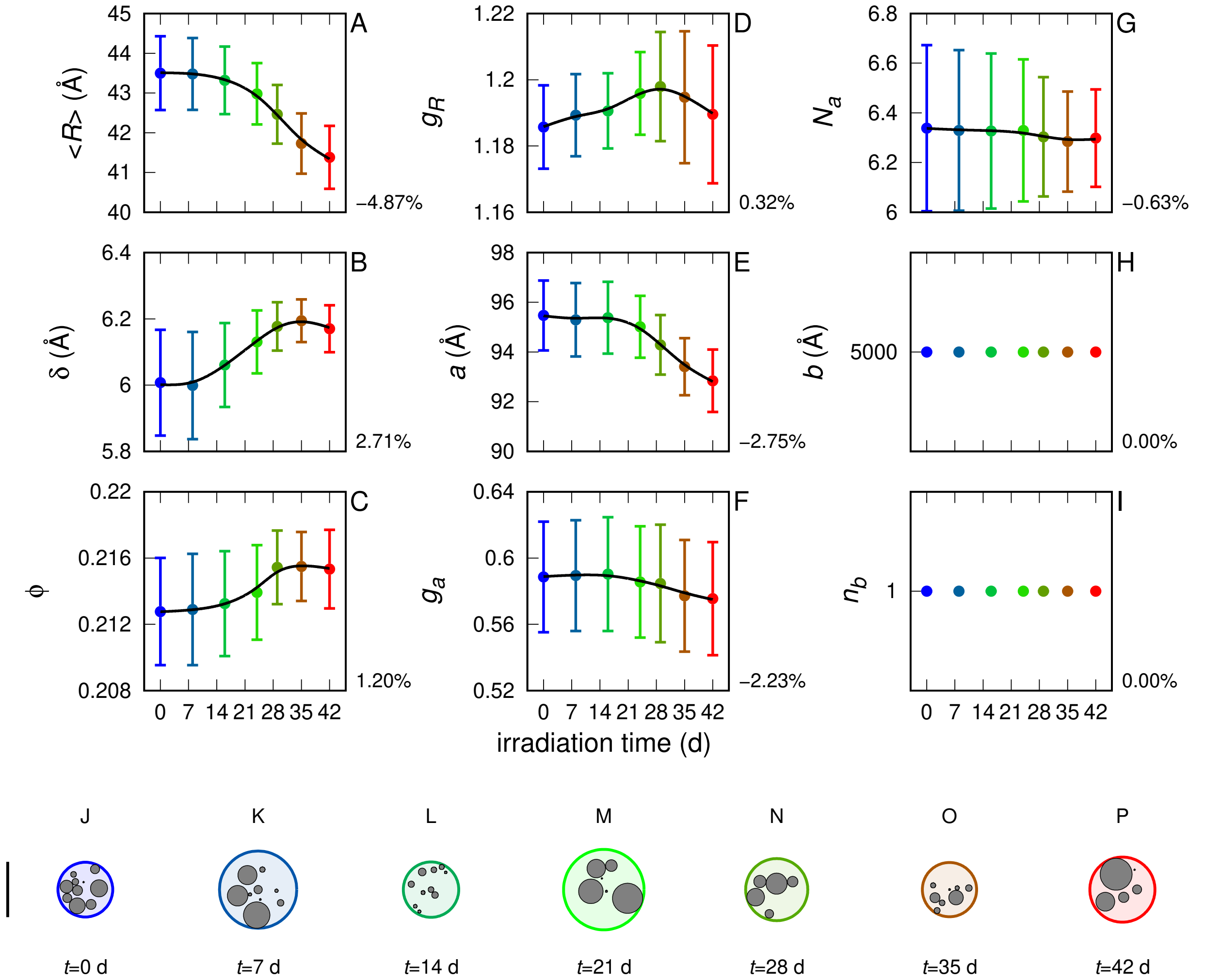

S22 Fig: **Fitting parameters from SAXS analysis of MFs of polyester in FW**. Smooth black curves among the points have been obtained with cubic splines weighted with uncertainties of the parameters. In the lower right corner of each panel, the percentage ratio between the difference from the final value ($t = 42$ d) and the initial value of the parameter ($t = 0$ d), relative to the initial value, is shown. Panels J-P: representation of the average MF cross section according to the fitting parameters. The small gray circles, representing the nanofibers, have been drawn with a radius sampled by the fitted Gaussian poly-dispersed distribution and in a position sampled according to the bidimensional para-crystal fitting parameters a, g_a and N_a. The vertical black line on the left of panel J represents the length of 1000 Å.

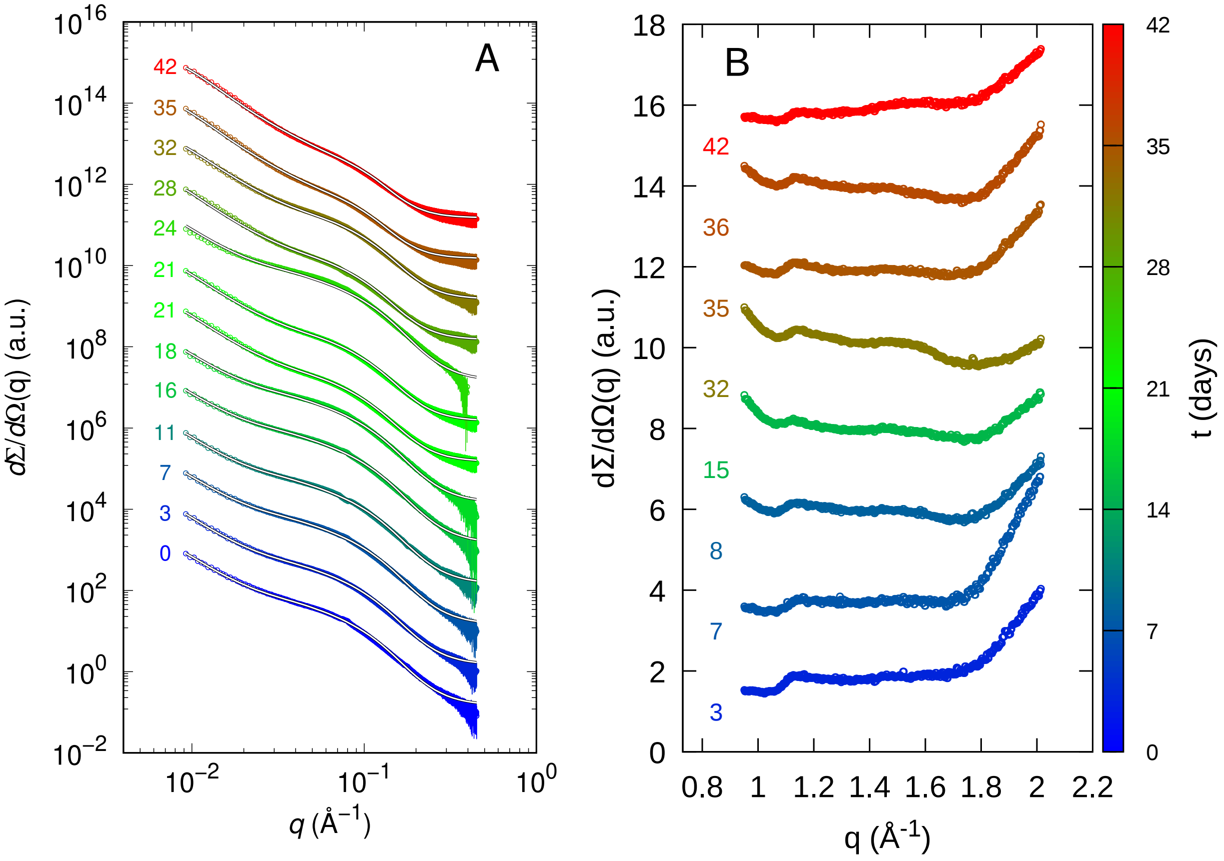

S23 Fig: **SAXS and WAXS curves of linen MFs in ASW**. The color of the data points corresponds to the vertical color scale, which indicates the UV irradiation time. A) The solid black and white lines represent the best fits obtained using GENFIT. Starting from the bottom, each curve is multiplied by a factor of 10 relative to the one below it, for clarity. B) The black and white lines correspond to the best fits obtained using GSAS. The experimental curves (shown without error bars for clarity) and their respective fits are presented, with the diffuse background, determined from the fit, subtracted from each. Thin vertical black lines indicate the Bragg reflections of the space group. Thick vertical black lines, with the corresponding Miller indices indicated, mark the positions of the main peaks. The curves are vertically offset by a factor of 2 for clarity.

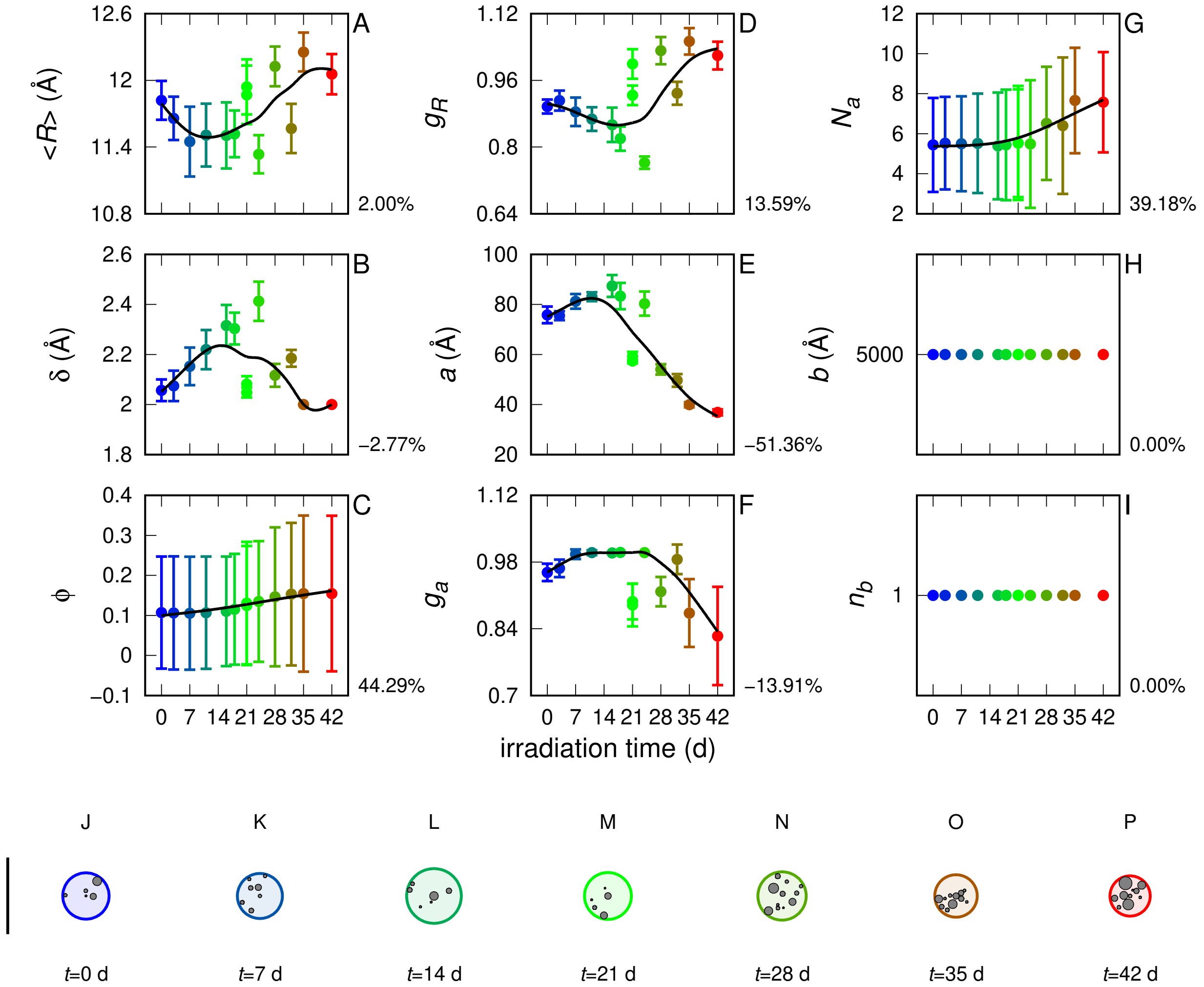

S24 Fig: **Fitting parameters from SAXS analysis of MFs of linen in ASW**. Smooth black curves among the points have been obtained with cubic splines weighted with uncertainties of the parameters. In the lower right corner of each panel, the percentage ratio between the difference from the final value ($t = 42$ d) and the initial value of the parameter ($t = 0$ d), relative to the initial value, is shown. Panels J-P: representation of the average MF cross section according to the fitting parameters. The small gray circles, representing the nanofibers, have been drawn with a radius sampled by the fitted Gaussian poly-dispersed distribution and in a position sampled according to the bidimensional para-crystal fitting parameters a, g_a and N_a. The vertical black line on the left of panel J represents the length of 1000 Å.

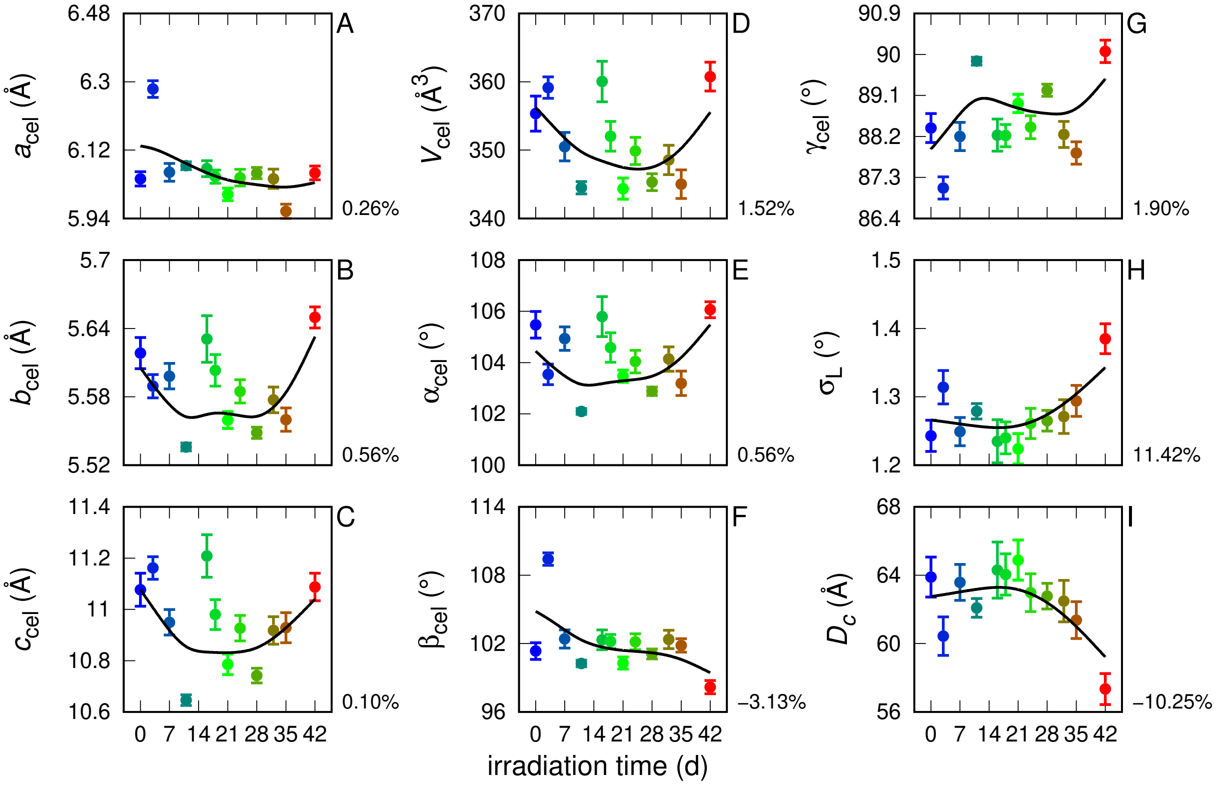

S25 Fig: **Fitting parameters from WAXS analysis of MFs of linen in ASW**. Smooth black curves among the points have been obtained with cubic splines weighted with uncertainties of the parameters. In the lower right corner of each panel, the percentage change between $t = 0$ d and $t = 42$ d, relative to the initial value, is reported.

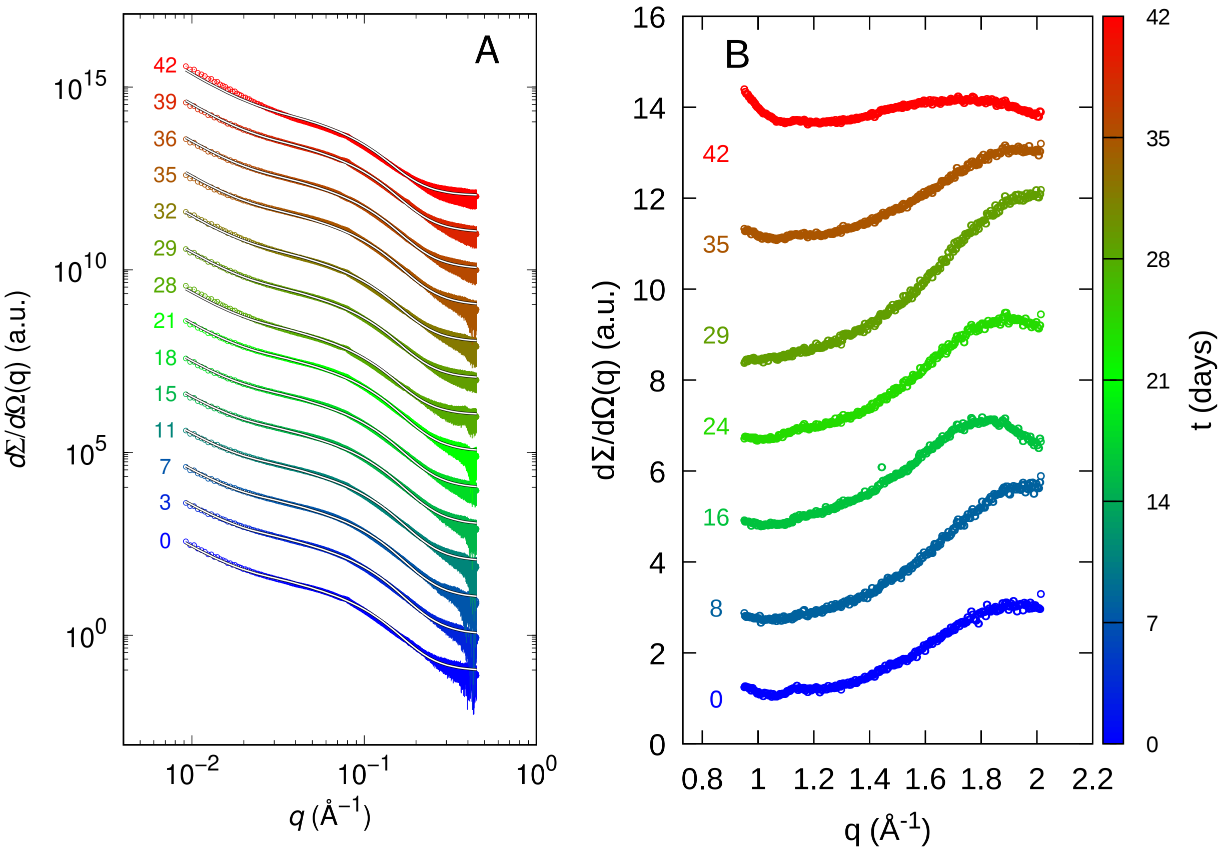

S26 Fig: **SAXS and WAXS curves of linen MFs in FW**. The color of the data points corresponds to the vertical color scale, which indicates the UV irradiation time. A) The solid black and white lines represent the best fits obtained using GENFIT. Starting from the bottom, each curve is multiplied by a factor of 10 relative to the one below it, for clarity. B) The black and white lines correspond to the best fits obtained using GSAS. The experimental curves (shown without error bars for clarity) and their respective fits are presented, with the diffuse background, determined from the fit, subtracted from each. Thin vertical black lines indicate the Bragg reflections of the space group. Thick vertical black lines, with the corresponding Miller indices indicated, mark the positions of the main peaks. The curves are vertically offset by a factor of 2 for clarity.

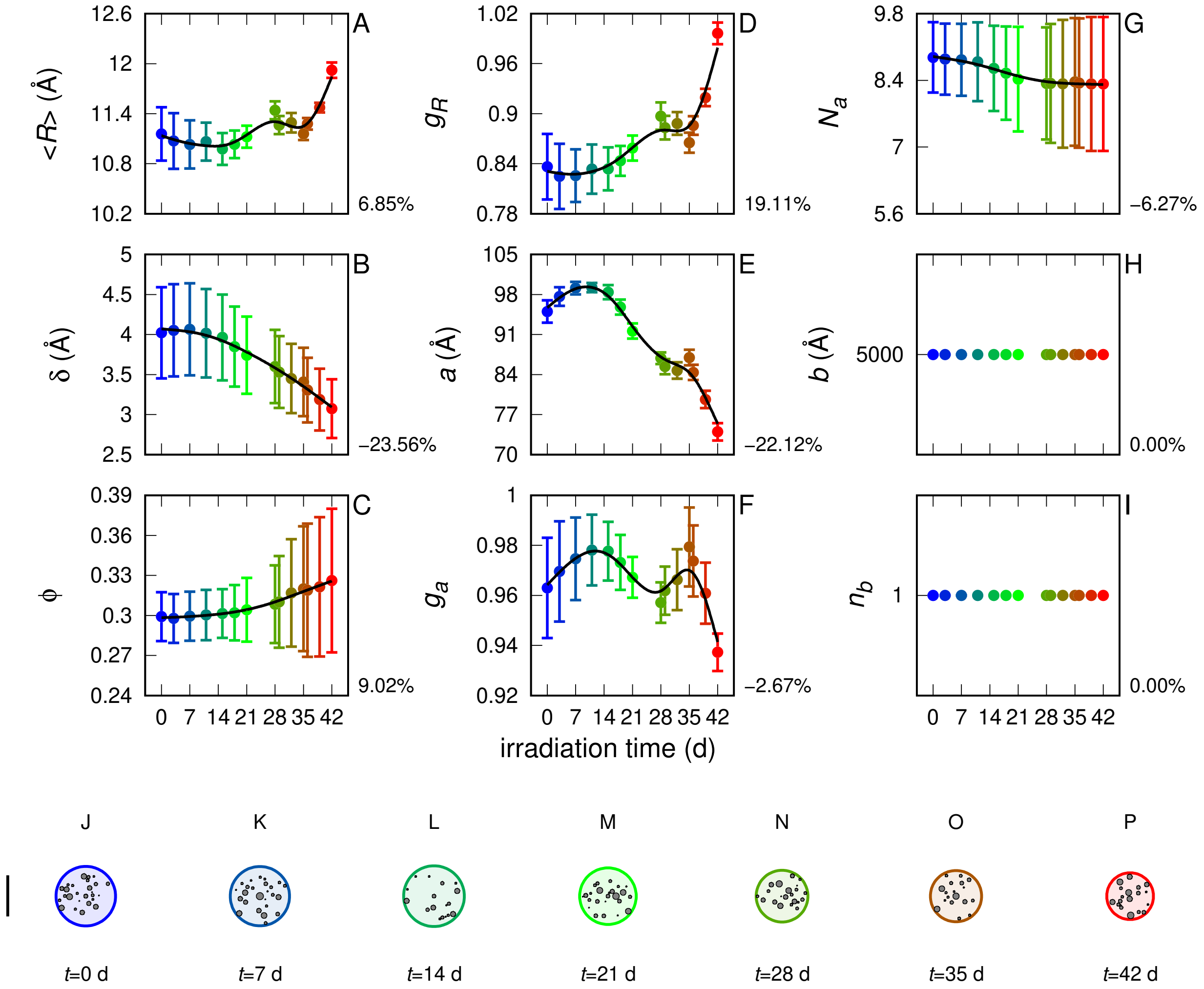

S27 Fig: **Fitting parameters from SAXS analysis of MFs of linen in FW**. Smooth black curves among the points have been obtained with cubic splines weighted with uncertainties of the parameters. In the lower right corner of each panel, the percentage ratio between the difference from the final value ($t = 42$ d) and the initial value of the parameter ($t = 0$ d), relative to the initial value, is shown. Panels J-P: representation of the average MF cross section according to the fitting parameters. The small gray circles, representing the nanofibers, have been drawn with a radius sampled by the fitted Gaussian poly-dispersed distribution and in a position sampled according to the bidimensional para-crystal fitting parameters a, g_a and N_a. The vertical black line on the left of panel J represents the length of 1000 Å.

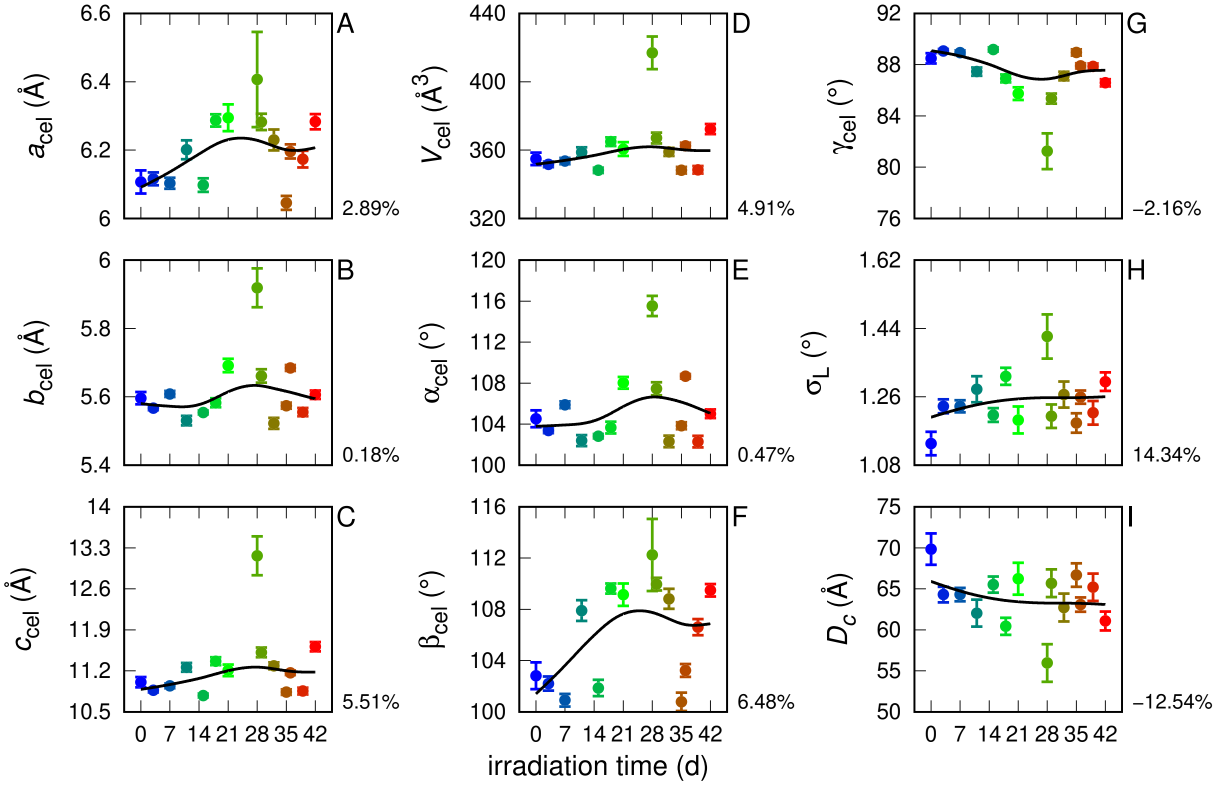

S28 Fig: **Fitting parameters from WAXS analysis of MFs of linen in FW**. Smooth black curves among the points have been obtained with cubic splines weighted with uncertainties of the parameters. In the lower right corner of each panel, the percentage change between $t = 0$ d and $t = 42$ d, relative to the initial value, is reported.

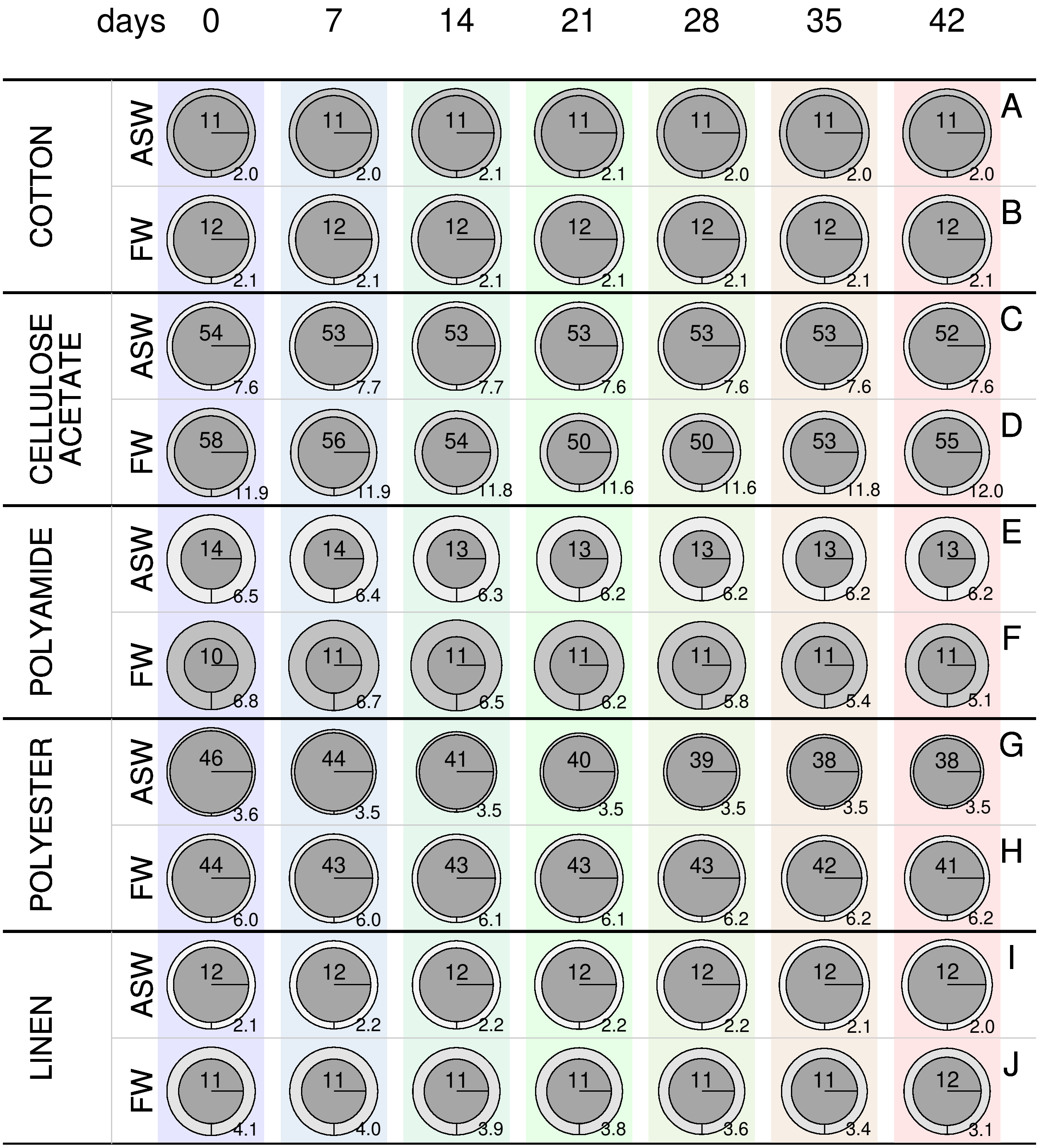

S29 Fig: **Core-shell cross sections of microfibrils during UV irradiation**. Cross-sectional views of the core-shell structure of microfibrils, derived from SAXS fitting parameters, for the five investigated MFs in ASW (panels A, C, E, G, and I) and in FW (panels B, D, F, H, and J). The radius of the inner core ($<R>$) and the shell thickness (δ) are indicated in Å at the top and bottom right corners of each image, respectively. The ratio between the gray intensity of the shell and that of the core reflects the fitting parameter ϕ.

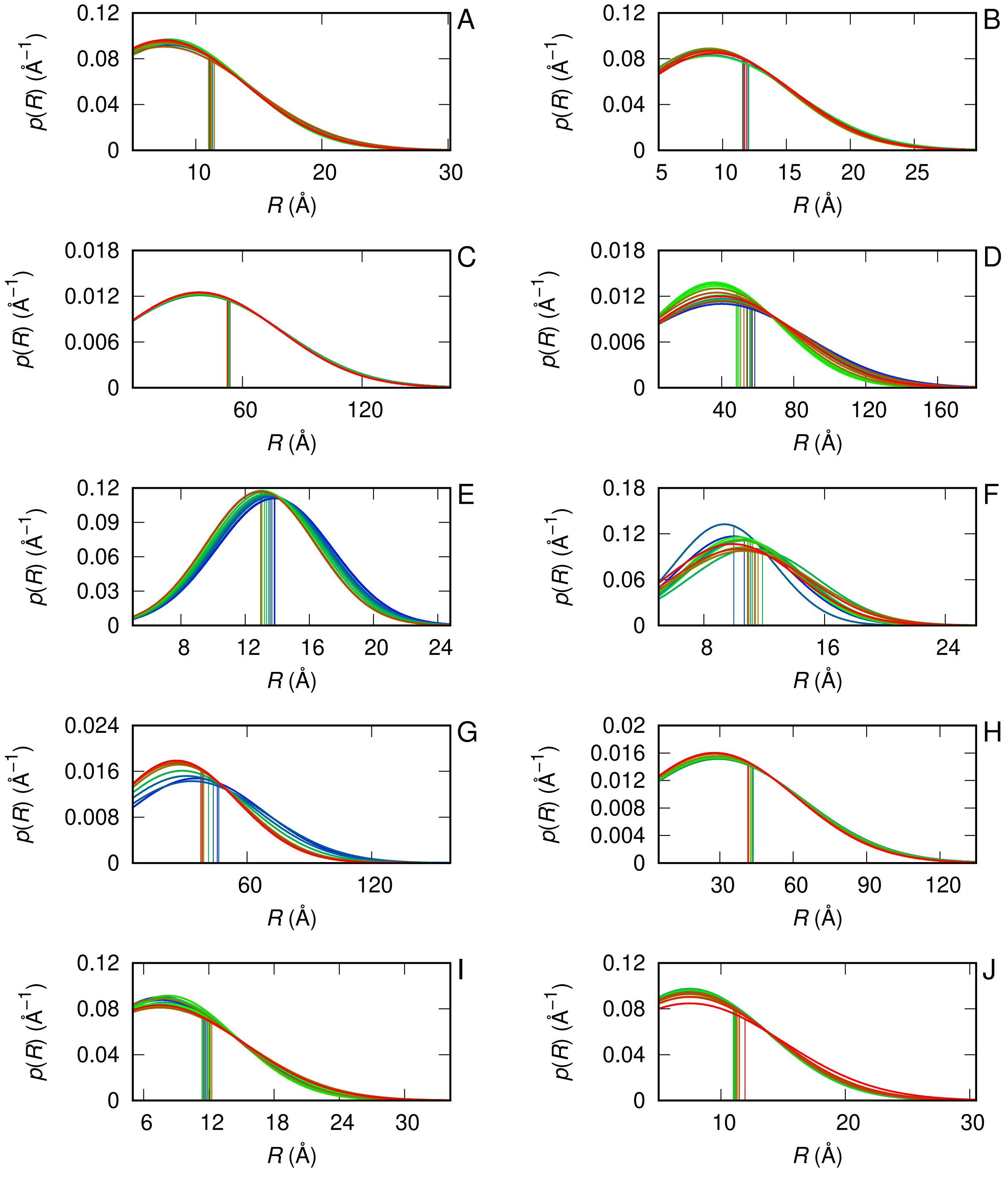

S30 Fig: **Core radius probability distribution function**. Plot of the functions $p(R)$ derived from SAXS fitting parameters, for the five investigated MFs in ASW (left panels) and in FW (right panels). The average radius of the inner core ($< R >$) is reported as vertical lines. Color codes depends on the UV irradiation time according to the palettes reported in Fig 2.
